# Complete chloroplast genome sequence of *Dryopteris fragrans* (L.) Schott and the repeat structures against the thermal environment

**DOI:** 10.1038/s41598-018-35061-8

**Published:** 2018-11-09

**Authors:** Rui Gao, Wenzhong Wang, Qingyang Huang, Ruifeng Fan, Xu Wang, Peng Feng, Guangming Zhao, Shuang Bian, Hongli Ren, Ying Chang

**Affiliations:** 1 Laboratory of Plant Research, College of Life Science, Northeast Agricultural University, Harbin, Heilongjiang Province 150030 P. R. China; 2 Virus-free Seedling Research Institute, Heilongjiang Academy of Agricultural Sciences, Harbin, Heilongjiang Province 150086 P. R. China; 3 Institute of Natural Resources and Ecology, Heilongjiang Academy of Science, Harbin, Heilongjiang Province 150040 P. R. China; 4 Medicine Key Laboratory of Chinese Materia Medica (Ministry of Education), Heilongjiang University of Chinese Medicine, Harbin, Heilongjiang Province 150040 P. R. China

## Abstract

*Dryopteris fragrans* (L.) Schott is a fern growing on the surface of hot rocks and lava. It is exposed to sunlight directly and bears local hot environment. We sequenced the complete nucleotide sequence of its chloroplast (cp) genome. The cp genome was 151,978 bp in length, consisting of a large single-copy region (85,332 bp), a small single-copy region (31,947 bp) and a pair of inverted repeats (17,314 bp). The cp genome contained 112 genes and 345 RNA editing sites in protein-coding genes. Simple sequence repeats (SSRs) and long repeat structure pairs (30–55 bp) were identified. The number and percent of repeat structures are extremely high in ferns. Thermal denaturation experiments showed its cp genome to have numerous, dispersed and high GC percent repeat structures, which conferred the strongest thermal stability. This repeat-heavy genome may provide the molecular basis of how *D. fragrans* cp survives its hot environment.

## Introduction

The chloroplast (cp) is a plant-specific and vital organelle that serves as the site of photosynthesis by converting light energy into chemical energy. This organelle is also involved in other biochemical processes, including sugar, amino acid, lipid, vitamin, starch and pigment syntheses, sulfate reduction and nitrogen metabolism^1–3^. The cp genome is typically circular and 120 to 160 kbp in size. Most cp genomes possess a similar structure. They typically contain a large single-copy region (LSC) and a short single-copy region (SSC) that are separated by two inverted repeats (IRs). The gene order and content of cp genomes are generally highly conserved, and the substitution rate in the cp DNA is less than that in nuclear DNA^4,5^. On the other hand, given their highly conserved sequences, similar structures and stable maternal heredity, cp genomes are a valuable and ideal resource for plant phylogenetics, population genetics, species identification and genetic engineering^6^. Gain and loss of function genes in the cp genome cause extensions or contractions, respectively, thus explaining genome size variations, which also reflect species differentiation events^1^.

Since the complete cp genome sequences of tobacco and liverwort were published^7,8^, an increasing number of seed plant cp genomes have been sequenced and reported^8^. With the development of next-generation sequencing (NGS), the cost of sequencing has been reduced, and the technique has become faster. NGS has the advantage of providing extremely high yield and accurate data on complete cp genomes. The number of sequenced cp genomes from various plants is increasing quickly. However, most complete current cp genome studies have focused on seed plants. Although ferns are a major group of plants, there are only 60 ferns for which complete cp genomes have been reported^9,10^. Other studies about ferns have been based on partial sequences or fragments of cp genomes^11^.

*Dryopteris fragrans* (L.) Schott is a perennial fern that grows on the surfaces of rocks and lava. It belongs to the Dryopteridaceae family and is found in the Far East, Europe and North America in small communities^12^. The Wudalianchi (N48°30′–48°51′, E126°00′–126^o^25′, Altitude 295–315 m) in Heilongjiang Province marks the centre of its distribution in China^13^. Its biotope is significantly different from that of other ferns. Most ferns prefer to grow in shady, warm and moist places. If those ferns are placed in dry and hot environments or exposed to ultraviolet (UV) ray for a short time, they quickly become crinkly and wilt or die. However, *D. fragrans* is exposed to sunlight and the dry surfaces of black rocks and lava directly. At the same time, it must endure strong UV rays and high surface temperatures of approximately 70 °C in summer. Therefore, this is a special species with superior stress resistance in ferns. This fern possesses special mechanisms to help it live in its severe environment. Previous studies of this species have mostly focused on its secondary metabolites and related genes^14–26^. Although lots of research has been performed on this species, its ability to survive in severe environments has largely been ignored. In our previous study, we partly sequenced its genome and obtained some contigs of its cp genome, which attracted our attention. Because the cp is the energy transducer for plants, it can induce changes in the external environment directly and react quickly. Studying the complete cp genome of *D. fragrans* may provide useful information about its superior heat resistance.

Here, we sequenced and annotated the complete cp genome of *D. fragrans*, and its cp genome features and structures were compared with related species. RNA editing sites were predicted by PREP in protein-coding genes and validated by transcripts. Simple sequence repeats (SSR) and long repeat structures were identified in this cp genome. The long repeat structures were extremely abundant compared with other species, and most were located in the intergenic spacer (IGS) region, which exhibited high GC content repeat structures and which may enhance cp genome stability. The thermal denaturation experiment showed that the *D. fragrans* cp genome exhibited strong thermal stability. These data would provide useful information and contribute to a better understanding of how this special fern lives in harsh environments. Furthermore, it will also be helpful in the study of secondary metabolism, genetic engineering, physiology and evolution within ferns and other species in the future.

## Results

### Chloroplast genome assembly and validation

Quantitative real-time polymerase chain reaction (qRT-PCR) result showed that the *rbcL* was detected in isolated cpDNA samples, while *actin 6*, the nuclear specific gene, was not detected (Supplemental Fig. 1). It showed that the cpDNA samples were pure. The sequencing run generated 2,740,440 raw reads, totaling 822,132,000 bases, with an average read length of 300 bp from the *D. fragrans* cp genome. A total of 2,262,910 clean reads with an average read length of 184.56 bp were *de novo* assembled into 31 contigs. The average sequence coverage depth of each nucleotide on the cp genome was 105X . A maximum scaffold size of 143,707 bp that spanned most of the small and large single copy region (SSC and LSC) and the entire inverted repeat (IR) region was generated. Because the IR region had double the coverage compared with the remaining scaffold, it was used twice in the complete cpDNA sequence. We submitted the annotated cp genome sequence of *D. fragrans* under GenBank accession number KX418656.2.

### Chloroplast genome features and comparison

**Figure 1:**
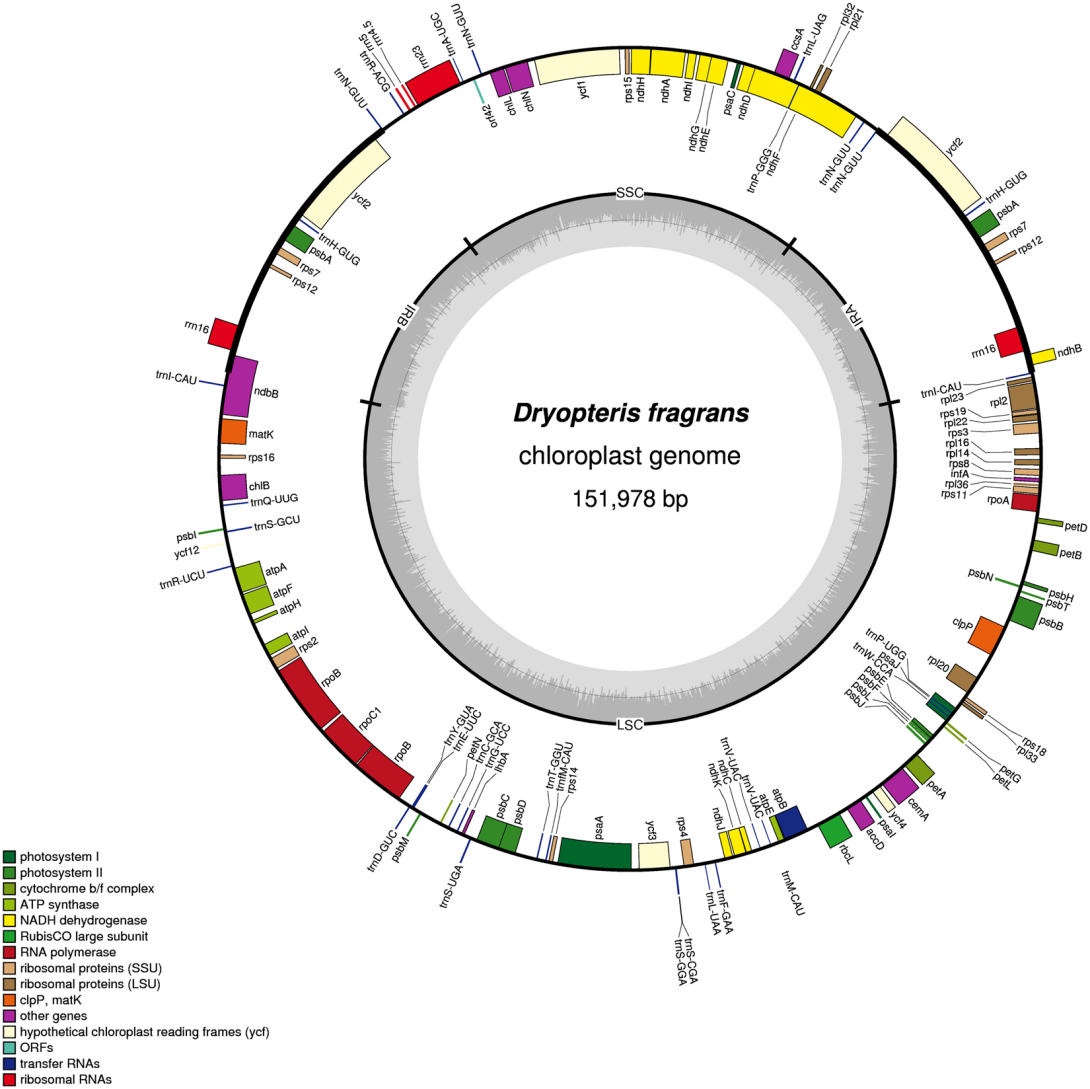
The mapped *D. fragrans* (L.) Schott circular chloroplast genome. Genes presented outside of the outer circle are transcribed in a clockwise manner, and those inside are transcribed in a counter-clockwise manner. Functional categories of genes are colour-coded. The dashed area in the inner circle indicates the GC content of the chloroplast genomes.

**Figure 2:**
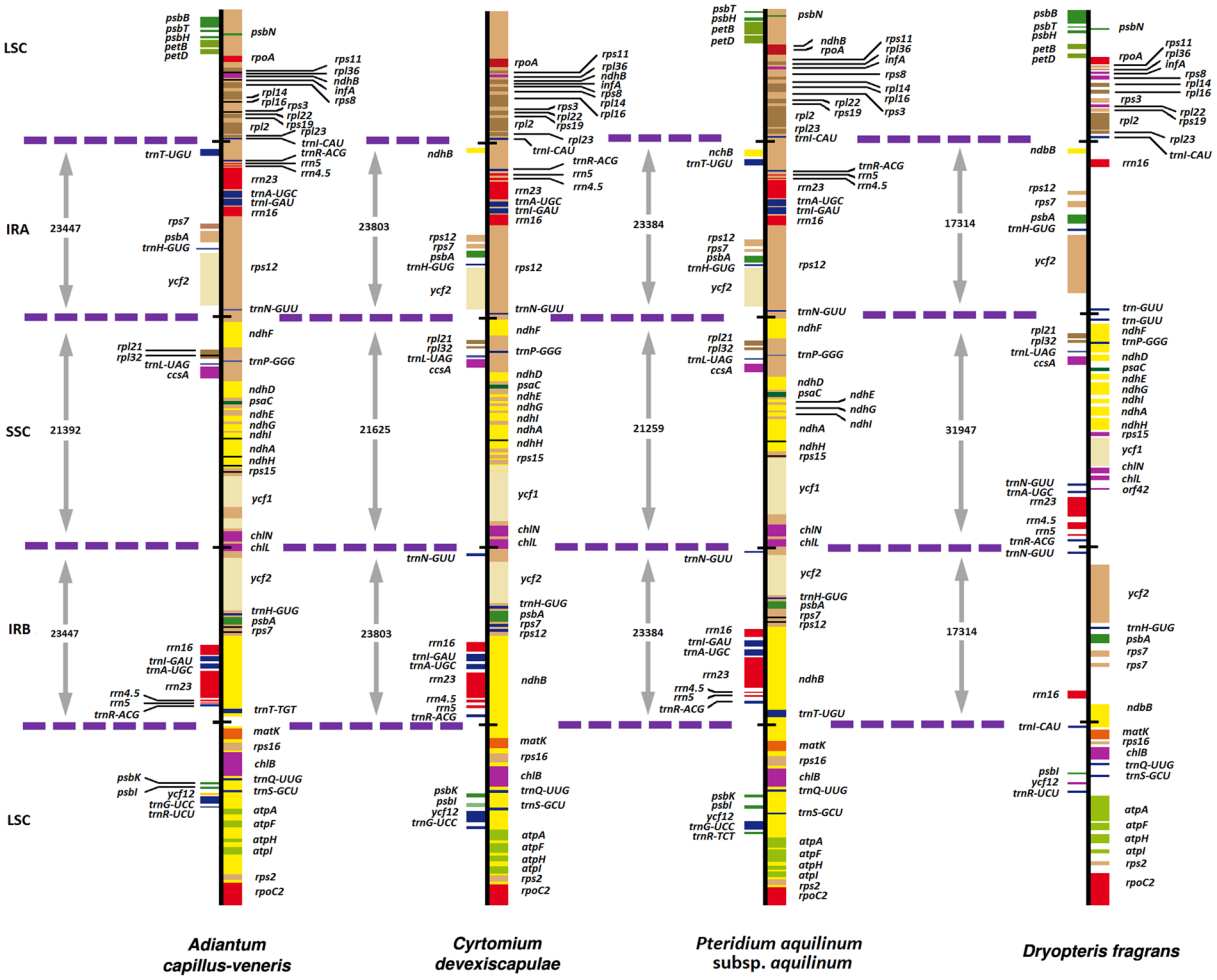
Comparison of gene order and content in the LSC, IR, and SSC regions among four cp genomes. Compared with other species, the IR length of *D. fragrans* is shorter and its SSC is the longest. The *D. fragrans* cp genome lost *ndhF* and gained *orf42, trnR-ACG, rrn5, rrn4.5, rrn23, trnA-UGC* and *trnN-GUU* in SSC. The *trnR-ACG*, *rrn5*, *rrn4.5*, *rps12* and *trnI-GUG* were lost in the IR regions, while *ndhB* was truncated in the IRA. The *psbK* and *trnG-UCC* were lost in IRB-LSC.

The cp genome of *D. fragrans* was 151,978 bp in length, with a typical quadripartite structure (Fig. 1). It included a pair of IRA and IRB of 17,314 bp separated by one SSC and one LSC of 31,947 bp and 85,332 bp, respectively. The *D. fragrans* cp genome contained 112 genes (Table 1), including 4 ribosomal RNA genes; 26 transfer RNA genes, and 22 genes encoding ribosomal subunits, of which 12 encoded the small subunit and 10 for the large subunit. It also included 3 genes encoding DNA-directed RNA polymerases, and 44 genes dedicated to photosynthesis, of which 11 encoded subunits of the NAD(P)H-quinone oxidoreductase, 4 encoded the photosystem I complex, 13 encoded the photosystem II complex, 6 encoded the cytochrome b/f complex, 6 encoded different subunits of the ATP synthase and 1 encoded the large chain of the ribulose bisphosphate carboxylase (*rbcL*). Three genes encoded the dark-operative protochlorophyllide oxidoreductase subunits; 5 genes (*ycf1, 2, 3, 4, 12*) were dedicated to open reading frames; 2 were detected to protease; 1 encoded a translational initiation factor and 5 for other proteins. Among them, 14 genes contained introns, including *psaA, atpF, ndhA, ndhB, ndhE, ndhF, ndhG, rpl2, rpl20, rpoB, rpoC1, cemA, clpP* and *ycf3* (Table 1). Compared with *Adiantum capillus-veneris*, *Pteridium* subsp. *aquilinum* and *Cyrtomium devexiscapulae*, the *D. fragrans* cp genome gained *orf42, trnR-ACG, rrn5, rrn4.5, rrn23, trnA-UGC and trnN-GUU* in SSC. In addition, *trnR-ACG*, *rrn5*, *rrn4.5*, *rps12* and *trnI-GUG* were lost in IRs, and *ndhB* was truncated in IRA. The *psbK* and *trnG-UCC* were lost in IRB-LSC (Fig. 2). The GC content of the cp genome was 43.15%. The IR region was the highest (44.18%), followed by the LSC (42.70%) and the SSC (43.26%). The GC contents in rRNA genes (55.75%) and tRNA genes (55.46%) were higher than those in protein coding regions (44.06%). The comparison of cp genome size, GC content, gene number and order is listed in Table 2. The *D. fragrans* cp genome did not contain tRNA for the amino acid codons Lys. The codon usage frequency was listed in Table 3. Among these codons, 987 (3.57%) encoding for glutamate and 166 (0.60%) for cysteine, were the most and the least amino acids codons, respectively.

**Table 1:** Genes present in the *D. fragrans* chloroplast genome.

Category for genes	Group of genes	Name of genes
Genes for photosynthesis	Photosystem I	*psaA* ^*#*^ *, C, I, J*
	Photosystem II	psbA*, B, C, D, E, F, H, I, J, L, M, N, T
	Cytochrome b/f complex	*petA, B, D, G, L, N*
	ATP synthase	*atpA, B, E, F*^#^*, H, I
	Rubisco	*rbcL*
	NADH oxidoreductase	*ndhA*^#^*, B^*#*^*, C, D, E*^#^*, F^*#*^*, G*^#^*, H, I, J, K
	Chlorophyll biosynthesis	*chlB, L, N*
Self-replication	Large subunit ribosomal proteins	*rpl2* ^*#*^ *, 14, 16, 20* ^*#*^ *, 21, 22, 23, 32, 33, 36*
	Small subunit ribosomal proteins	rps2, 3, 4, 6, 7*, 8, 11, 12*, 14, 15, 18, 19
	DNA dependent RNA polymerase	*rpoA, B*^#^*, C1^*#*^
	Ribosomal RNAs	rrn4.5, 5, 16*, 23
	Transfer RNAs	trnA-UGC, trnC-GCA, trnD-GUC, trnE-UUC, trnF-GAA, trnG-UCC, trnH-GUG*, trnI-CAU*, trnL-UAA*, trnL-UAG, trnM-CAU**, trnfM-CAU, trnN-GUU**, trnP-GGG, trnP-UGG, trnQ-UUG, trnR-ACG, trnR-UCU, trnS-CGA, trnS-GCU, trnS-GGA, trnS-UGA, trnT-GGU, trnV-UAC*, trnW-CCA, trnY-GUA
Other genes	Other proteins	*accD, orf42, cemA* ^*#*^ *, ccsA, lhbA*
	Protease	*clpP* ^*#*^ *, matK*
	Translational initiation factor	*infA*
Genes of unknown function	Open Reading Frames (ORF, ycf)	*ycf1, 2, 3* ^*#*^ *, 4, 12*

*Double copies; **Four copies; ^#^intron.

**Table 2:** The characters of chloroplast genomes selected from 21 Pteridophyta, 1 Gymnospermae, 1 Monocot and 1 Dicot.

	Total Length (bp)	GC content (%)	LSC Length (bp)	SSC Length (bp)	IR Length (bp)	Number of genes*	Protein gene	rRNA gene	tRNA gene
*Dryopteris fragrans*	151987	43.15	85332	31947	17314	112	82	4	26
*Adiantum capillus-veneris*	150568	42	82282	21392	23447	117	84	4	29
*Alsophila spinulosa*	156661	40.43	86308	21623	24365	117	85	4	28
*Angiopteris evecta*	153901	35.48	89709	22086	21053	121	85	4	32
*Athyrium anisopterum*	151284	43.98	21771	23123	83267	117	84	4	29
*Athyrium opacum*	150979	43.69	21779	23052	83096	117	84	4	29
*Austroblechnum melanocaulon*	150202	43.68	21604	22621	83356	117	84	4	29
*Cyrtomium devexiscapulae*	181684	42.33	82453	21625	23803	117	84	4	29
*Deparia lancea*	151011	43.9	21832	23087	83005	117	84	4	29
*Diplazium dushanense*	150179	43.2	21844	23044	83182	117	84	4	29
*Homalosorus pycnocarpos*	152159	43.2	21810	23203	83943	116	84	4	28
*Huperzia lucidula*	154373	36.2	104088	19657	15314	120	87	4	29
*Isoetes flaccida*	145303	39.9	91862	27205	13118	118	82	4	32
*Macrothelypteris torresiana*	151130	43.1	21852	23048	83182	117	84	4	29
*Matteuccia struthiopteris*	151003	44.3	21760	23243	82757	116	84	4	28
*Onoclea sensibilis*	148395	44.4	21813	22113	82356	112	82	4	26
*Osmundastrum cinnamomeum*	142812	40.2	100294	22300	10109	121	84	4	33
*Pseudophegopteris aurita*	149917	43.07	21472	22807	82831	118	84	4	30
*Psilotum nudum*	138829	36.03	84617	16304	18954	118	81	4	33
*Pteridium aquilinum* subsp*. aquilinum*	152362	41.5	84335	21259	23384	117	84	4	29
*Woodwardia unigemmata*	153717	43.21	82387	21556	24887	117	84	4	29
*Ginkgo biloba*	156945	39.60	99223	22254	17734	122	83	4	35
*Arabidopsis thaliana*	154478	36.30	84170	26264	17780	112	79	3	30
*Oryza sativa*	134525	39.00	80592	12355	20799	126	92	4	30

*The reduplicate genes in the cp genome were considered only once.

**Table 3:** Codon usage and codon-anticodon recognition pattern for tRNA in *D. fragrans* cp genome.

Amino acid	Codon	Number	RSCU	tRNA	Amino acid	Codon	Number	RSCU	tRNA
Phe	UUU	776	1.12		Tyr	UAU	549	1.17	
	UUC	608	0.88	*trnF-GAA*		UAC	387	0.83	*trnY-GUA*
Leu	UUA	669	1.43	*trnL-UAA*	His	CAU	355	1.15	
	UUG	621	1.33			CAC	261	0.85	*trnH-GUG*
	CUU	429	0.92		Gln	CAA	580	1.29	*trnQ-UUG*
	CUC	312	0.67			CAG	318	0.71	
	CUA	453	0.97	*trnL-UAG*	Asn	AAU	781	1.3	
	CUG	314	0.67			AAC	418	0.7	*trnN-GUU*
Ile	AUU	942	1.42		Lys	AAA	841	1.32	
	AUC	501	0.76			AAG	437	0.68	
	AUA	544	0.82		Asp	GAU	809	1.4	
Met	AUG	568	1	*trnM-CAU*		GAC	344	0.6	*trnD-GUC*
Val	GUU	536	1.28		Glu	GAA	987	1.38	*trnE-UUC*
	GUC	298	0.71			GAG	443	0.62	
	GUA	580	1.38	*trnV-UAC*	Cys	UGU	197	1.09	
	GUG	267	0.64			UGC	166	0.91	*trnC-GCA*
Ser	UCU	553	1.33		Trp	UGG	400	1	*trnW-CCA*
	UCC	407	0.98	*trnS-GGA*	Arg	CGU	367	1.17	*trnR-ACG*
	UCA	516	1.24	*trnS-UGA*		CGC	198	0.63	
	UCG	358	0.86	*trnS-CGA*		CGA	346	1.1	
Pro	CCU	327	1.01			CGG	225	0.72	
	CCC	392	1.21	*trnP-GGG*	Ser	AGU	425	1.02	
	CCA	366	1.13	*trnP-UGG*		AGC	240	0.58	*trnS-GCU*
	CCG	213	0.66		Arg	AGA	487	1.55	*trnR-UCU*
Thr	ACU	485	1.31			AGG	258	0.82	
	ACC	372	1	*trnT-GGU*	Gly	GGU	627	1.29	
	ACA	357	0.96			GGC	281	0.58	
	ACG	272	0.73			GGA	642	1.33	*trnG-UCC*
Ala	GCU	655	1.52			GGG	388	0.8	
	GCC	358	0.83		TER	UAA	55	1.05	
	GCA	417	0.97	*trnA-UGC*		UAG	54	1.03	
	GCG	294	0.68			UGA	48	0.92	

### SSRs and repeat structures in the *D. fragrans* chloroplast genome

**Figure 3:**
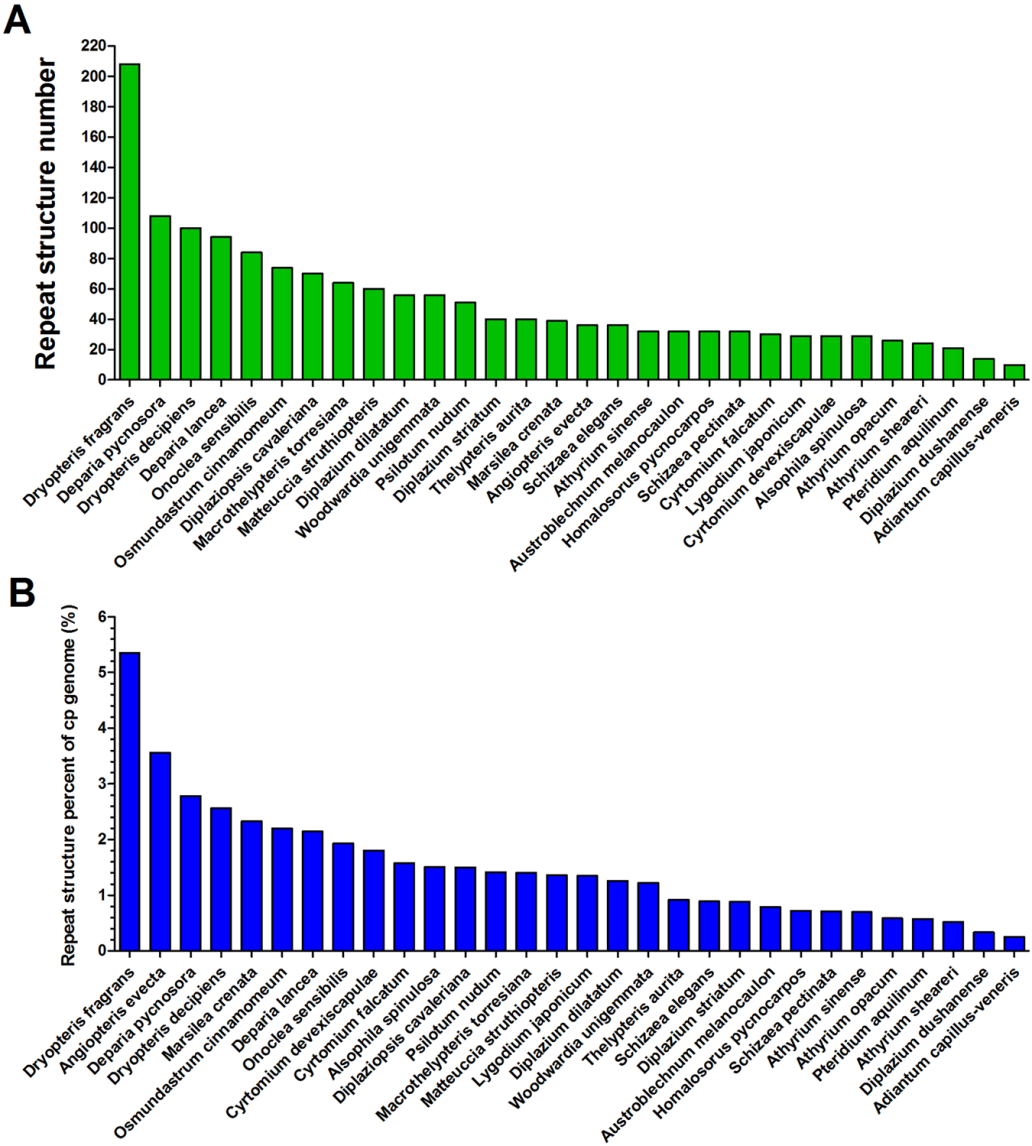
Comparisons of repeat structure number and percent within 30 ferns. The sizes of the repeats are set at a repeat minimal length of ≥33 bp and maximal length of ≤55 bp with a Hamming distance of 3. The number and percentage of the repeat structures from 29 ferns were compared with those of *D. fragrans*. (**A**) The number of repeat structure in the *D. fragrans* cp genome was compared to that of 29 ferns. *D. fragrans* possesses the most repeat structures; (**B**) The percent of repeat structures in the *D. fragrans* cp genome was compared to that of 29 ferns. *D. fragrans* possesses the highest repeat structure percent (5.351%).

The MISA detected 44 SSRs in the *D. fragrans* cp genome (Supplemental Table 2), including 41 homopolymers and 3 dipolymers. Tetrapolymers, pentapolymers, and hexapolymers were not found. Sixteen SSRs were exclusively composed of A or T bases, 27 SSRs were G or C bases, and 1 was an AG base. Most of the bases were G or C bases, except for the AG dipolymer. All of these SSRs were located in the IGS, and none were located in protein-coding genes. Repeat analysis by REPuter, with the criterion of a copy size of ≥30 bp or longer and a sequence identity ≥90%, identified 80 forward, 1 reverse and 23 palindromic repeat structure pairs from 30 to 55 bp. Repeat lengths of 30 to 32 bp were most common (27.40%). A total of 53 repeat pairs were found in the coding regions, of which 6 were located in the transfer RNA genes. The remaining 151 repeat pairs were located in the IGSs (Supplemental Table 3). In addition, one of the longest repeat structure (55 bp) overlapped with the longest SSR sequence (18 bp G mononucleotide sequence) (Supplemental Table 3). The average GC content of repeat structures was 43.04%, with a maximum of 63.64% and minimum of 30%. To compare the number of repeat structures with that of other fern species, we extracted correlative sequences from 29 ferns to determine the number of repeat structures of different lengths. Repeat structures were abundantly distributed in the *D. fragrans* cp genome, and this species contained the most repeat structures among ferns (Fig. 3A). Compared with the other 29 genomes, the *D. fragrans* cp genome had the highest percentage of repeat structures (5.351%). It was higher than the other species by a factor of 1.50 to 21.28 (Fig. 3B). Thus, *D. fragrans* was rich in repeat structures.

### RNA editing

The transcripts obtained by PCR were used to verify the RNA editing sites predicted by PREP. The PREP prediction results showed that there were 438 RNA editing sites in protein-encoding genes, corresponding to 338 codon changes. All editing events were of the C-to-U variety. Among them, 96 non-synonymous mutations were found at the first position of the codon, while the remaining mutations were found at the second position, and none were found at the third position. However, in the transcript validation results, there were 345 RNA editing sites in the *D. fragrans* cp genome (Supplemental Table 4). In all, 88 mutations occurred in the first position of the codon, 208 at the second position, and 49 at the third position. The C-U mutations were the most common, reaching 305 (88.41%) mutations, followed by U-C 13 (3.77%), G-A 8 (2.32%), A-G 7 (2.03%), A-C 3 (0.87%), C-G 2 (0.58%), G-U 2 (0.58%), U-A 2 (0.58%), U-G 2 (0.58%) and G-C 1 (0.29%) (Supplemental Fig. 2). There were 318 codon changes, including 33 synonymous mutations and 285 nonsynonymous mutations. The majority of editing sites were predicted in the *ndhF* gene (130629-123964, 41 editing sites), followed by the *atpB* gene (72943-71462, 21 editing sites). The conversions of amino acids included 119 hydrophilic to hydrophobic changes (H to Y, S to L, S to F, and T to M) and 105 hydrophobic to hydrophobic changes (L to F, A to V, P to S, R to W and P to L). The codons that turned into leucine (Leu) were the most common, accounting for 125 changes (39.31%). The number of RNA editing sites (TCA (S)-TTA (L)) were the most frequent (37 editing sites), followed TCG(S)-TTG(L) (28 editing sites) and CCA(P)-CTA(L) (21 editing sites).

### Comparison of cpDNA thermal stability

**Figure 4:**
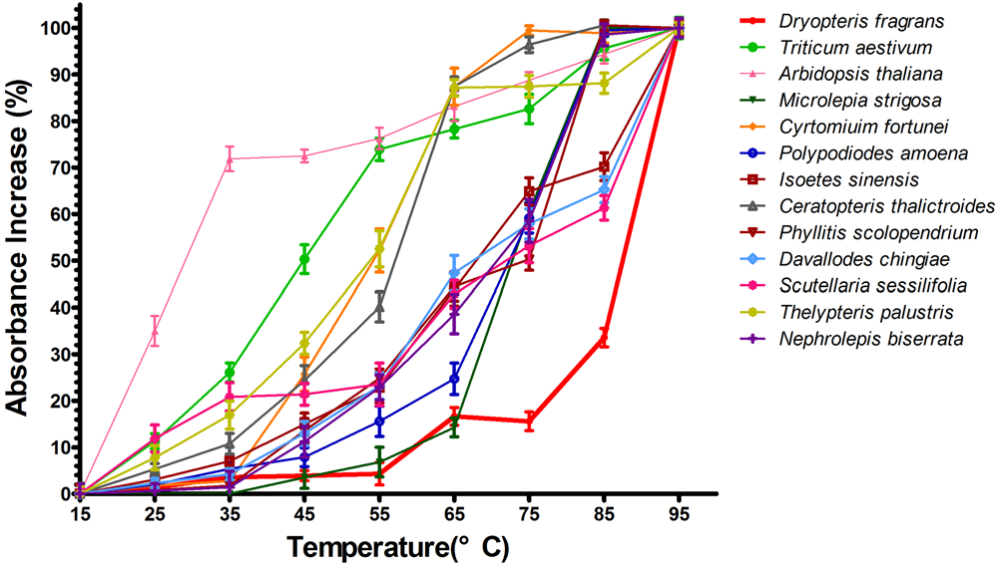
Percent of absorbance increases the variations of cpDNA in the thermal denaturation. The absorbance increases of 8 plants, including 6 ferns, 1 dicotyledon and 1 monocotyledon, were compared with *D. fragrans* (red). *D. fragrans* shows considerable stability against heat.

To confirm that repeat structures with high GC content contributed enhanced the cp genome stability of *D. fragrans*, the thermal denaturation for all species cp genomes were completed. In the denaturation experiment, the absorbance of all samples increased with elevated temperatures (Fig. 4). The percentage increase of all ferns was under or approximately 20% at 35 °C. Some rose quickly, such as Arabidopsis, wheat, and *T. palustris*. Most samples began to go up quickly at 35 °C and sharply rose at 55 °C. Almost all cp genomes could not bear 75 °C and their absorbance increased greatly. However, only *D. fragrans* maintained it from beginning to 55 °C and changed slightly from 65 °C to 75 °C. Even at 85 °C, *D. fragrans* still kept the lowest value compared to the others. It showed that its cp genome could cope with heating.

## Discussion

DOGMA is the most popular software for cp genome annotation and is used widely^27–30^. This software can detect protein-coding genes, rRNA, and tRNA quickly. However, it also has drawbacks in detecting genes, because its ability to detect introns is not very sensitive. In our work, DOGMA annotated 110 genes but did not detect genes with intron(s). We performed another software analysis using MAKER-P. It detected 9 genes (*ndhF*, *rpl21*, *rps6*, *cemA*, *ccsA*, *lhbA*, *matK*, *ycf1* and *ycf12*) that were not annotated by DOGMA. It also identified 14 genes containing intron(s), and their positions were also corrected (Table 1). The genes in the fern cp genomes are different from those of seed plants, although both of them are vascular plants. This made DOGMA perform not very well in ferns and produced incorrect result. The MAKER-P showed an advantage in the detection of protein-coding genes and introns. Thus, annotation for fern cp genomes requires the use of different software programs.

The typical size of fern cp genome is 131 to 168 kb^31,32^, and the *D. fragrans* cp genome is within this range. The gene number and order are largely similar to the cp genomes of ferns, but there are some differences among species. The genome size variation is mostly due to length variation in the IR and the SSC regions^28^. Some IR expansions/contractions are observed within species. Compared to other ferns, the IR regions of the *D. fragrans* cp genome lost a 4033 bp sequence, including *trnR-ACG, rrn5, rrn4.5, rrn23, trnA-UGC* and *trnN-GUU*. This sequence was located in IRs of other fern cp genomes. However, this sequence in the *D. fragrans* cp genome was moved into SSC. Thus, these genes did not exist as two copies in the *D. fragrans* cp genome. It is possible that the fern reduced the expression of these genes. The phenomenon causes the *D. fragrans* cp genome to contain the longest SSC and shorter IRs (Table 2). Alhough synteny and inversions are important, the structure of the *D. fragrans* cp genome does not show obvious changes. It is consistent with results of Xiang *et al*.^9^. Furthermore, overlapped genes are not notable (Fig. 1), which would reduce the cp genome usage. Thus, the *D. fragrans* cp genome has more intergenic sequences, leading to a more dispersed gene distribution and increasing the sequence length. These findings suggest that the fern cp genome chooses reduces the overlapping genes but extends the intergenic sequences. Thus, genes are more independent and sequence utilization is more specific.

RNA editing is an important post-transcriptional process in cps and is thought to be functionally significant^33^. In our work, the phenomenon was obvious in protein-coding genes. There were great disparities between the results of PREP and transcript validation. The number of RNA editing sites and codon transformations in the PREP results were far more than those of the validation. This result indicates that there may be some deficiencies in PREP, though the prediction results conformed to the number and quantity of predicted variations in general. This may be because the PREP database is not abundant enough, especially for ferns. Moreover, the result shows that transcript verification is necessary for RNA editing site prediction. In seed plant cps, a conversion from C-to-U is the most predominant form^34^, and reverse U-to-C editing is the opposite in seed plants^35^. Most editing events in the *D. fragrans* cp genome were C-to-U (88.4%) events. At the same time, its C-to-U transition is the most frequent type of base change. It has been reported that the excess of C-to-U RNA editing developed in early stages of vascular plant evolution^36^. Our results support this view. On the other hand, the number and percent of codons transitioning to Leu were the highest in the *D. fragrans* cp genome. It is similar with those of *Adiantum capillus-veneris*, though the genetic distance between *A. capillus-veneris* and *D. fragrans* is long in the fern clade. Leu biosynthesis occurs in cp and plays an important role in photosynthesis-related metabolism^37,38^. Both species account for a heavily used Leu codon, suggesting they have a great need for Leu. Their level of RNA editing is more than ten times that of any other vascular plant examined across an entire cp genome^39^. This reflects the fact that RNA editing occurs in different fern species and may play a major role in fern cp and cp genome processing.

Simple sequence repeats (SSRs) ranging in length from 1–6 or or more base pairs, also known as microsatellites and short tandem repeats (STRs), are important genetic molecular markers for population genetics^40,41^ and are widely used for plant genotyping^42,43^. In this work, there were 44 SSRs in the *D. fragrans* cp genome. The number of GC SSRs was more than the number of AT SSRs. This finding contrasts with the view that cp SSRs are generally composed of short polyadenine (poly A) or polythymine (poly T) repeats and rarely contain tandem guanine (G) or cytosine (C) repeats^44^. On the other hand, the number and percent of repeat structure (30–55 bp) in the *D. fragrans* cp genome were far more than other species (Fig. 3). This is the first time that a fern species has been shown to contain a considerable number of repeat structures. It shows that its cp genome is rich in repeat structures. At the same time, most repeat structures were located in every IGS dispersedly, and the GC percentages of most repeat structures were higher than the average value (43.04%). This indicates that the dispersed repeat structures probably play a key role in maintaining cp genome stability. *D. fragrans* may increase the IGS number and length of inserted repeat structures with a high active GC content. Previous has work suggested that repeat structures are very important for sequence rearrangement and variation in cp genomes by preventing illegitimate combinations and slipped-strand mispairing^1,45,46^. Our results could support this point of view. Furthermore, these repeat structures have also became a part of the intergenic sequences between genes, resulting in the independence of each gene. This feature allows for the selective expression of genes.

Wudalianchi was formed in great volcanic eruption. Its physiognomy is mainly consist of alkaline basalt^47^. This is a kind of black volcanic rock with low specific heat capacity (0.84 kJ/(kg·K)) and small thermal conductivity. The basalt would absorb lots of heat quickly under direct and long sunshine in summer. It could result in high surface temperature easily and form a local hot environment in the range of basalt geomorphology. The temperature of basalt surface in summer often reaches 70 °C. On the other hand, the basalt topography cools quickly at night. *D. fragrans* grows on the exposed basalt surface and is exposed to large temperature fluctuations between day and night. Most ferns cannot endure such high temperature and dramatic temperature changes, but *D. fragrans* is a rare fern and is highly resistant to heat. In the results mentioned above, our study revealed this fern possesses the greatest number of repeat structures, with a high GC percentage, among all ferns studied. The three hydrogen bonds between GC are stronger than the two between AT, such that the GC percentage determines the strength of the DNA double chain (i.e., loose or tight). Some researchers have noted that the higher the level of GC content, the more stable the structure of the genome DNA^48^. We speculate that these repeat structures with high GC content may allow the fern to cope with heat and large temperature differences. Thus, we performed a heat denaturation experiment to compare the cpDNA thermal stability of ferns species and closely related species from different habitats and families, including Nephrolepidaceae, Thelypteridaceae, Pteridaceae, Davalliaceae, Aspleniaceae, Polypodiaceae, Dryopteridaceae, Dennstaedtiaceae, Parkeriaceae and Isoetaceae. Arabidopsis and wheat showed the most obvious changes in the denaturation experiment. This indicates that their cpDNA is very sensitive to heat. *S. sessilifolia*, *T. palustris, C. fortunei, I. sinensis* and *C. thalictroides* changed earlier and largely under 45 °C. The habitats of these five species are swamps or humid underforest environments. Their heat resistance was weak. *D. chingia, N. biserrata*, *P. scolopendrium, M. strigosa* and *P. amoena* showed smaller variations and similar heat stability between 35–45 °C. However, most of them could not survive at temperatures over 45 °C and began to rise significantly with the increase of temperature (Fig. 4). *D. chingia, P. amoena* and *M. strigosa* are saxicolous ferns in forest. Their cpDNA showed heat resistance and their thermal stability was limited. In addition, there were great differences between *D. fragrans* and *C. fortunei* in terms of their thermal stability, although both of them belong to the Dryopteridaceae family. This suggests that great differences exist within species of the same family, which is caused by different environments. These results support the speculation that a considerable number of dispersed repeat structures with a high GC content (43.04%) enhance *D. fragrans* cpDNA thermal stability and maintain its structure in the face of thermal changes. It is one of molecular basis of *D. fragrans* in response to severe environments. It also provided a new scope for understanding the environmental adaption mechanisms of plants.

## Methods

### Plants, cp DNA extraction, sequencing and assembly

Fresh leaves of *D. fragrans* from Wudalianchi, Heilongjiang Province were collected and frozen in liquid nitrogen after cleaning. Professor Baodong Liu of Harbin Normal University provided the leaves of *Nephrolepis biserrata*, *Polypodiodes amoena*, *Isoetes sinensis, Cyrtomium fortunei, Phyllitis scolopendrium*, *Davallodes chingiae, Scutellaria sessilifolia*, *Microlepia strigosa*, and *Thelypteris palustris*. The *Arabidopsis thaliana*, wheat (*Triticum aestivum* L.) and *Ceratopteris thalictroides* were collected in our lab. The cp isolation methods were modified based on previous methods^49–51^. Five grams of complete leaves from all species were picked and rinsed. The leaves were then crushed in liquid nitrogen and added to a separation solution (0.33 M D-Sorbitol, 50 mM Tris-HCl pH 7.6, 5 mM MgCl_2_, 10 mM NaCl, 2 mM EDTA, 2 mM D-sodium erythorbat and 0.2% beta-mercaptol) for grinding. The cell suspensions were filtered, and the filtrate was centrifuged at 1000 rpm for 10 min to eliminate large-sized cell fragments. The supernatant was collected and centrifuged at 4000 rpm for 10 min to separate the cp. We obtained cps and extracted pure cpDNA using the CTAB-based method^52^. Every DNA sample was treated with RNase. To assess the contamination of the nuclear genome, nuclear special gene, *actin6*, and cp special gene, *rbcL*, were selected to performed qRT-PCR. The specific primer pairs or degenerate primer pairs were designed based on special sequence or homologous sequences (Supplemental Table 1). LineGene 9620 instrument (HANGZHOU BIOER TECHNOLOGY Co., LTD. China) and TransStart Green qPCR SuperMix (TRANSGEN BIOTECH, China) were used for detection. The qRT-PCR program was set as follows, 5 min at 95 °C, 40 cycles of 15 s at 95 °C, and 30 s at 60 °C. The cpDNA samples of *D. fragrans* cp genome were sequenced using Illumina technology on HiSeq. 2000 at Genewiz (China). To improve the Illumina sequence read quality and accuracy of the sequences, we performed Trimmomatic (version 0.30)^53^ to optimize the processing for filtering the adaptor sequence. The software SSPACE (version 3.0)^54^, GapFiller (version 1.10)^55^ and Velvet (version 1.2.10)^56^ were used to examine the raw reads and assemble them into contigs and scaffolds with default parameters. There were some gaps left after assembly. To finish the assembly of the whole genome, gaps were filled by PCR. PCR reactions were performed in a total volume of 20 μL containing 6 μL of deionized sterile water, 10 μL of EasyTaq Mix buffer, 1 μL of each primer at 10 pmol/μL (TransGen Biotech, Beijing, China) and 2 μL of cp DNA. PCR products were purified and sequenced by Bio-Serve (Harbin, China). All primers used for gap filling are listed in Supplemental Table 1.

### Chloroplast genome annotation and comparative analyses

Gene location and annotations of the *D. fragrans* cp were performed using the Dual Organellar GenoMe Annotator (DOGMA) (http://dogma.ccbb.utexas.edu)^57^ and MAKER-P^58,59^, including protein-coding and rRNA and tRNA genes. All genes, rRNAs, and tRNAs were identified using the plastid/bacterial genetic code. The predicted annotations were verified using Chloroplast Genome DB (http://chloroplast.cbio.psu.edu/)^60^ and Blast^61^. tRNAscan-SE was used to identify the tRNAs^62^. Codon usage and relative synonymous codon usage (RSCU) were calculated by CodonW 1.4.2 (http://codonw.sourceforge.net)^63^. The annotated sequence was submitted to GenBank. The circular gene map of the *D. fragrans* cp was generated using OGDRAW^64^.

The GC%, LSC, SSC, IR regions, gene number and length of complete genome of the *D. fragrans* cp genome were compared to the cp genomes from *Adiantum capillus-veneris* (NC_004766)*, Osmundastrum cinnamomeum* (NC_024157)*, Cyrtomium devexiscapulae* (KT599100)*, Woodwardia unigemmata* (KT599101)*, Alsophila spinulosa* (NC_012818)*, Psilotum nudum* (NC_003386), *Pteridium aquilinum* subsp. *aquilinum* (NC_104348)*, Angiopteris evecta* (NC_008829)*, Isoetes flaccida* (NC_014675)*, Huperzia lucidula* (NC_006861)*, Athyrium anisopterum* (NC_035738.1), *Athyrium opacum* (NC_035841.1), *Austroblechnum melanocaulon* (NC_035840.1), *Deparia lancea* (NC_035844.1), *Diplazium dushanense* (NC_035851.1), *Homalosorus pycnocarpos* (NC_035855.1), *Macrothelypteris torresiana* (NC_035858.1), *Matteuccia struthiopteris* (NC_035859.1), *Onoclea sensibilis* (NC_035861.1), *Pseudophegopteris aurita* (NC_035861.1), *Ginkgo biloba* (AB684440)*, Arabidopsis thaliana* (NC_000932) and *Oryza sativa* (NC_001320). Furthermore, we compared the borders, gene content and order of the LSC, SSC and IRs regions with those of *A. capillus-veneris, P. aquilinum* subsp. *aquilinum* and *C. devexiscapulae*.

### Examination of the repeat sequences and RNA editing

MISA, a microsatellite identification tool (http://pgrc.ipk-gatersleben.de/misa/misa.html), was used to detect SSRs^65^, with thresholds of mononucleotide repeats ≥10 bases, dinucleotide repeats ≥6 bases, tri- and tetranucleotide repeats ≥5 bases, and hexanucleotide or greater repeats ≥5 bases. The max distance between two SSRs was 100 base pairs. Based on these analyses, we identified the location of the SSRs. The REPuter program^66^ was used to assess long repeat sequences on the forward, reverse and palindrome sequences within the cp genomes. The sizes of the repeats were set at a repeat minimal length of ≥30 bp and a maximal length of ≤55 bp with a Hamming distance of 3. Furthermore, we selected 29 ferns, including *Adiantum capillus-veneris*, *Alsophila spinulosa*, *Angiopteris evecta*, *Athyrium opacum, A. sinense, A. sheareri*, *Austroblechnum melanocaulon, Cyrtomium falcatum*, *C. devexiscapulae*, *Deparia pycnosora, Diplaziopsis cavaleriana, Diplazium dilatatum*, *D. dushanense, D. lancea*, *D. striatum*, *Dryopteris decipiens, Homalosorus pycnocarpos*, *Lygodium japonicum*, *Macrothelypteris torresiana, Matteuccia struthiopteris*, *Marsilea crenata, Osmundastrum cinnamomeum*, *Onoclea sensibilis*, *Psilotum nudum*, *Pteridium aquilinum* subsp. *aquilinum, Schizaea elegans, S. pectinat, Thelypteris aurita* and *W. unigemmata* to calculate the long repeat sequences using the same parameters. We compared differences in the repeat numbers of different lengths from those ferns.

### Prediction and Transcript validation of RNA editing sites

The predictive RNA Editor for Plants (PREP) was used to predict potential RNA editing sites in protein-coding genes with a cutoff value of 0.8^67^. The protein-coding genes were *accD, atpA, atpB, atpI, ccsA, clpP, matK, ndhB, ndhD, ndhF, ndhG, petB, petD, petD, petL, psaI, psbB, psbE, psbF, psbL, rpoA, rpoB, rpoC1, rps14, rps2, rps2* and *ycf3*. Total RNA was isolated from leaves using Tiangen™ Plant Total RNA Kit (China). The quality and concentration of RNA samples were examined by agarose gel electrophoresis and spectrophotometer analysis, respectively. The first-strand cDNA was prepared with 3 μg of total RNA using the TransScript One-Step gDNA Removal and cDNA Synthesis SuperMix Kit (Transgen Biotech, China). Primer pairs for each gene were designed based on extracted gene sequences and are listed in Supplemental Table 5. The PCR was carried out as follows: 5 min at 95 °C, 30 cycles of 30 s at 95 °C, 30 s at 56–63 °C, 60 s at 72 °C and 10 min at 72 °C. The PCR products were sequenced at HaiGene (Harbin, China). The sequences were aligned with extracted gene sequences. The RNA editing sites validated by PCR were collected and compared to the PREP results.

### Thermal denaturation and renaturation of cp genomes

DNA denaturation produces hyperchromic effect. The isolated cpDNA from all species was used. For denaturation, the absorbance increase could reflect the thermal stability of cpDNA by gradient thermal treatment. All cpDNA from species were dissolved in Tris-EDTA (TE) buffer. The concentration was adjusted to 50 ng/mL. Absorbance at 260 nm was used to monitor the denaturation processes, and the TE buffer was used as a blank control. Each sample was treated in different temperatures water bath (25 °C, 35 °C, 45 °C, 55 °C, 65 °C, 75 °C, 85 °C, 95 °C), and each temperature treatment went for 10 min. The absorbance of the initial temperature treatment was set as A_0_ (15 °C), and the value of highest temperature treatment was set at A_max_ (95 °C). The cp DNA was heated to 95 °C in order to melt the DNA completely and determine limit values for cp genomes, such that the standards for each cp genome can be provided. The formula to determine the increase percentages of the absorbance increase (AI) of the hyperchromic effects as: AI = (A_temp_ - A_0_)/(A_max_ - A_0_) × 100%. The recorded data were repeated 3 times and collected to calculate.

## Electronic supplementary material

Supplementary file
