# Supplementary material for: Complete chloroplast genome sequence of Dryopteris fragrans (L.) Schott and the repeat structures against the thermal environment
Source: Sci Rep. 2018 Nov 9;8:16635. doi: 10.1038/s41598-018-35061-8 (PMC6226466; doi:10.1038/s41598-018-35061-8)
Supplement: Supplementary file 1 — Supplementary file [file 41598_2018_35061_MOESM1_ESM.doc]

**Complete chloroplast genome sequence of *Dryopteris fragrans* (L.) Schott and the repeat structures against thermal environment**

Rui Gao^1^, Wenzhong Wang^1,2^, Qingyang Huang^3^, Ruifeng Fan^4^, Xu Wang^1^, Peng Feng^1^, Guangming Zhao^1^, Shuang Bian^1^, Hongli Ren^1^, Ying Chang^1^*

^1^ Laboratory of Plant Research, College of Life Science, Northeast Agricultural University, Harbin, Heilongjiang Province, 150030, P. R. China

^2^ Virus-free Seedling Research Institute, Heilongjiang Academy of Agricultural Sciences, Harbin, Heilongjiang Province, 150086, P. R. China

^3^ Institute of Natural Resources and Ecology, Heilongjiang Academy of Science, Harbin, Heilongjiang Province, 150040, P. R. China

^4^ Medicine Key Laboratory of Chinese Materia Medica (Ministry of Education), Heilongjiang University of Chinese Medicine, Harbin, Heilongjiang Province, 150040, P. R. China

* Correspondence: changying@neau.edu.cn; Tel.: +86 0451 55190410; Fax: +86 0451 55190413

E-mail : changying@neau.edu.cn

**Supplemental Table 1. PCR primers designed for filling gaps and identification of nuclear genome DNA and chloroplast DNA.**

| **Genes and gaps** | **Primer name** | **Sequence** |
| --- | --- | --- |
| *actin6* for *D. fragrans* | Dfactin6-F | TAGCCTCCTTTCCCGCTCCCG |
|  | Dfactin6-R | ACCTCCAAGTCCGACGCCACA |
| *rbcL* for *D. fragrans* | DfrbcL-F | CTGCGGTAGCTGCGGAATCCTC |
|  | DfrbcL-R | GTATGTGATCTCCACCGGACATGCG |
| Degenerate primer pair | Dp-actin-F | CACACNGTBCCHATCTAHGARGGBYAYGC |
|  | Dp-actin-R | GHSCVATVGTVATVABYTGBCCRTCWGG |
|  | Dp-rbcL-F | GCYGCRGTAGCKGCVGRATC |
|  | Dp-rbcL-R | TCYCTYTCVACYTGRATVCCRTGAGGNGG |
| *actin1* for Arabidopsis | Atactin1-F | ACCGGTGTGATGGTTGGGATG |
|  | Atactin1-R | TCCTCAGGAGCAACACGGAGC |
| *rbcL* for Arabidopsis | AtrbcL-F | TGCTACTGCGGGTACATGCGA |
|  | AtrbcL-R | TAACAGCGTGCATTGCACGGT |
| *actin* for wheat | Wactin-F | CCTCTCAACCCCAAGGCCAAC |
|  | Wactin-R | TGGAACAGTGTGGCTCACACC |
| *rbcL* for wheat | WrbcL-F | CTGGGGAAGACAGCCAATGGA |
|  | WrbcL-R | CTTGGATACCATGAGGCGGGC |
| 1-2 | 1'-2'F | CGACCAGTGCGACTTCCTGCC |
|  | 1'-2'R | CGAAACCCGGATGCGAAATCGAG |
| 2-3 | 2'-3'F | ACCCCATGGAGAGTTGGCTGC |
|  | 2'-3'R | AGCACCCGAAACGCGAGAATCT |
| 3-4 | 3'-4'F | AGTCGGGGTAGCAAAAGCCGC |
|  | 3'-4'R | AGGCGCTCCTCGAACCAATCA |
| 4-5 | 4'-5'F | AGGAGTTGAACCCGTGAATTCGCC |
|  | 4'-5'R | CGGGGTGATCTCGCAGTTCCT |
| 5-6 | 5'-6'F | AGGAAGAAGCGGGTCGGGAGA |
|  | 5'-6'R | GTGCGGCTGGATCACCTCCTT |
| 6-7 | 6'-7'F | AACAGCCGACCGCTCTACCGC |
|  | 6'-7'R | TGGAGAGGGTATACGACGCGGG |
| 7-8 | 7'-8'F | GGACACGCCCTCACACACTGG |
|  | 7'-8'R | TGTGCGTCGGATTTATCGGCCT |
| 8-1 | 8'-1'F | GCCAGCCTCTACGCGGTCTAC |
|  | 8'-1'R | CGACACCGTGGTTCGTAGCCA |

**Supplemental Table 2. List of simple sequence repeats (SSR) in the *D. fragrans* cp genome.**

| **Repeat unit** | **Length (bp)** | **Number of SSRs** | **Position & Location** |
| --- | --- | --- | --- |
| A | 10 | 4 | 1661 [IGS(*trnR-ACG, trnN-GUU*)]; 26837 [IGS(*chlB, trnQ-UUG*)]; 87914 [IGS(*rps12, clpP*)]; 128426 [IGS(*trnL-UAG, ndhD*)] |
|  | 11 | 1 | 93950 [IGS(*psbH, petB*)] |
| T | 10 | 6 | 618 [IGS(*trnR-ACG, trnN-GUU*)]; 22477 [IGS(*trnI-CAU, chlB*)]; 55545 [IGS(*psbD, trnT-GGU*)]; 89600 [IGS(*clpP, clpP*)]; 126433 [IGS(*trnN-GUU, rpl32*)]; 131752 [IGS(*psaC, ndhE*)] |
|  | 11 | 1 | 52824 [IGS(*trnS-UGA, psbC*)] |
|  | 12 | 1 | 50718 [IGS(*petN, trnC-GCA*)] |
|  | 13 | 1 | 132052 [IGS(*psaC, ndhE*)] |
| G | 10 | 4 | 70099 [IGS(*rrn16, ndhB*)]; 94118 [IGS(*trnQ-UUG, psbI*)]; 113477 [IGS(*trnV-UAC, trnV-UAC*)]; 123200 [IGS(*trnN-GUU, trnN-GUU*)] |
|  | 11 | 2 | 75456 [IGS(*rbcL*, *accD*)]; 17911 [IGS(*rrn16, ndhB*)] |
|  | 17 | 1 | 94356 [IGS(*psbH, petB*)] |
|  | 18 | 1 | 122733 [IGS(*trnN-GUU, trnN-GUU*)] |
|  | 21 | 1 | 123645 [IGS(trnN-GUU, rpl32)] |
| C | 10 | 6 | 10741 [IGS(*psbA, rps7*)]; 28936 [IGS(*trnS-GCU, ycf12*)]; 51239 [IGS(*trnC-GCA, trnG-UCC*)]; 134738 [IGS(*ndhI, ndhA*)]; 147228 [IGS(*trnN-GUU, orf42*)]; 147945 [IGS(*orf42, trnA-UGC*)] |
|  | 11 | 2 | 76528 [IGS(*accD, psaI*)]; 106306 [IGS(*ndhB, rrn16*)] |
|  | 12 | 4 | 24151 [IGS(*trnI-CAU, chlB*)]; 43298 [IGS(*rpoC1, rpoC1*)]; 98971 [IGS(*infA, rps8*)]; 146775 [IGS(*chlL*, *trnN*-*GUU*)] |
|  | 13 | 1 | 28674 [IGS(*trnS*-*GCU*, *ycf12*)] |
|  | 16 | 1 | 86684 [IGS(*rpl20, rps12_5end*)] |
|  | 19 | 2 | 34691 [IGS(*atpH, atpI*)]; 131899 [IGS(*psaC, ndhE*)] |
|  | 22 | 2 | 85956 [IGS(*rpl20, rps12_5end*)]; 86871 [IGS(*rpl20*, *rps12_5end*)] |
| AT | 8 | 1 | 56164 [IGS(*psbD, trnT-GGU*)] |
| TA | 7 | 1 | 70680 [IGS(*trnV-UAC, trnM-CAU*)] |
| AG | 6 | 1 | 83809 [IGS(*trnW-CCA, trnP-UGG*)] |

**Supplemental Table 3. List of long repeat structures in the *D. fragrans* cp genome.**

| **Size** | **Type** | **Start position** | **Repeat unit** | **Location1** | **Location2** |
| --- | --- | --- | --- | --- | --- |
| 55 | P | 122691^*^; 146774 | GCAATCCCATTTGAATGGAAGGGTACCCGAATCGTGGGAGAGGGGGGGGGGGGGG | IGS (*trnN-GUU, trnN-GUU*) | IGS (*trnN-GUU, rpl32*) |
| 54 | F | 85821; 86549 | GAAAATTATATTTTCGTCTGAGAAATCCATTTATTCCCGGGCTCTGCTATTCCC | IGS (*rpl20, rps12_5end*) | IGS (*rpl20, rps12_5end*) |
| 54 | P | 2067; 123049 | ATAAGTCATCTGGGGAGGGGACTAAATCCACAATTTTAGAAAGGATCTATTCCA | IGS (*trnN-GUU, ycf2*) | IGS (*trnN-GUU, orf42*) |
| 54 | F | 18678; 19993 | TAAGAGATTCACAACTGATGTTGCTCCCAGAACCTCCCTCGTGTATGTTTCAAA | IGS (*trnI-CAU, chlB*) | IGS (*ndhB, ndhB*) |
| 54 | P | 19993; 105494 | TTTGAAACATACACGAGGGAGGTTCTGGGAGCAACATCAGTTGTGAATCTCTTA | IGS (*ndhB, ndhB*) | IGS (*trnI-CAU, ndhB*) |
| 54 | F | 122105; 123049 | TGGAATAGATCCTTTCTAAAATTGTGGATTTAGTCCCCTCCCCAGATGACTTAT | IGS (*ycf2, trnN-GUU*) | IGS (*trnN-GUU, trnN-GUU*) |
| 53 | F | 132293; 133046 | GCAGTAGCTAACCCGGTGGCAGCCTCGGCAGCCGCAACGGCTATCACAAATAT | *ndhE* (reverse strand) | *ndhE* (reverse strand) |
| 53 | F | 85489; 86216 | CAGTAAGCGTCTGGAGTAGCTACTTGCGCGAGTGTCTTGCGATTCAGAAAAAC | *rpl20* (reverse strand) | IGS (*rpl20, rps12_5end* (reverse strand)) |
| 52 | F | 93110; 93728 | AAAGCTCAAACTATGATTAATTTGCATCAATTTACCATTAAAATTTCGTGAC | IGS (*psbH, petB*) | IGS (*rpl20, rps12_5end*) |
| 52 | F | 34643; 35080 | ATGGCTCAATTTAAATTTGGAGGGCCATGCGGGAATTATTAACCCCTCCCCC | IGS (*atpH, atpI*) | IGS (*atpH, atpI*) |
| 52 | F | 122802; 122838 | GTGTCGACAGTGTCGATAGTGTCGATAGTGTCGATAGTGTCGATAGTGTCGA | IGS (*trnN-GUU, trnN-GUU*) | IGS (*trnN-GUU, trnN-GUU*) |
| 51 | P | 123784; 146607 | TCCTACCTCATAGGAAGAAGTAATATCTTTGATAAATAGAAGTAGCAAAAA | IGS (*trnN-GUU, rpl32*) | IGS (*chlL, trnN-GUU*) |
| 51 | F | 48254; 48273 | GGTTTCGGGGGGCTCATTCAAGATTATTTAGAGCGGCGAACCAGCAGGAAA | *rpl20* (reverse strand) | *rpl20* (reverse strand) |
| 51 | F | 85633; 86360 | AATTTTCTCTTCCGGCTATTTCTATCTACATAAGCGCAAGCTAATGCCCTT | *rpl20* (reverse strand) | *rpl20* (reverse strand) |
| 51 | F | 85685; 86412 | TTTCCTGCTGGTTCGCCGCTCTAAATAATCTTGAATGAGCCCCCCGAAACC | *rpl20* (reverse strand) | *rpl20* (reverse strand) |
| 48 | F | 86761; 86947 | GCGCTTTTCAATTTTTGGAAGGTCGTTAGGTAGCTGCTTCATAATTTT | IGS (*rpl20, rps12_5end*) | IGS (*rpl20, rps12_5end*) |
| 48 | F | 122810; 122846 | AGTGTCGATAGTGTCGATAGTGTCGATAGTGTCGATAGTGTCGATAGT | IGS (*trnN-GUU, trnN-GUU*) | IGS (*trnN-GUU, trnN-GUU*) |
| 47 | F | 85859; 86587 | GGGCTCTGCTATTCCCCTAAATAATTTCGTTTCAATATTTCTTATTT | IGS (*rpl20, rps12_5end*) | IGS (*rpl20, rps12_5end*) |
| 47 | F | 85924; 86652 | AAATAGATATGCTGTGTTACACCGAACTGTACCCCCCCCCCCCCCCC | IGS (*rpl20, rps12_5end*) | IGS (*rpl20, rps12_5end*) |
| 46 | F | 94117; 94369 | GGGGGGGGGGTACATCGATAAGATTAAAAGATTTCCCAAGACGCTA | IGS (*psbH, petB*) | IGS (*psbH, petB*) |
| 46 | F | 85399; 86126 | TTATTTCTGGCCTCTCCGTCTAATCCGGAGAGGCTTACCGTCGCAT | IGS (*rps18, rpl20*) | IGS (*rpl20, rps12_5end*) |
| 45 | F | 151880; 151922 | TGAGAGATGAGAGATGAGAGATGAGAGATGAGAGATGAGAGATGA | IGS (*rrn5, trnR-ACG*) | IGS (*rrn5, trnR-ACG*) |
| 45 | P | 1933; 123164 | TTAATAGGCAGGGTCCTTGCCTTGGGGGTATTCGAGGGGGGGGGG | IGS (*trnN-GUU, orf42*) | IGS (*trnN-GUU, trnN-GUU*) |
| 45 | F | 1933; 147227 | CCCCCCCCCCTCGAATACCCCCAAGGCAAGGACCCTGCCTATTAA | IGS (*trnN-GUU, orf42*) | IGS (*trnN-GUU, ycf2*) |
| 45 | F | 122248; 123164 | TTAATAGGCAGGGTCCTTGCCTTGGGGGTATTCGAGGGGGGGGGG | IGS (*ycf2, trnN-GUU*) | IGS (*trnN-GUU, trnN-GUU*) |
| 45 | F | 122248; 147227 | TTAATAGGCAGGGTCCTTGCCTTGGGGGTATTCGAGGGGGGGGGG | IGS (*ycf2, trnN-GUU*) | IGS (*trnN-GUU, ycf2*) |
| 45 | F | 122682; 123598 | GCGGCGATAGCAATCCCATTTGAATGGAATGGTACCCGAATCGTG | IGS (*trnN-GUU, rpl32*) | IGS (*trnN-GUU, trnN-GUU*) |
| 45 | F | 85408; 86135 | GCCTCTCCGTTTAATCCGGAGAGGCTTACCGCCGCATGTCAATCT | IGS (*rpl20, rps12_5end*) | IGS (*rps18, rpl20*) |
| 44 | F | 122161; 123105 | ATTCGCTCATTTCAATCATTCAATACACCGGAATTGGAGGGGGG | IGS (*ycf2, trnN-GUU*) | IGS (*trnN-GUU, trnN-GUU*) |
| 44 | P | 122161; 147287 | CCCCCCTCCAATTCCGGTGTATTGAATGATTGAAATGAGCGAAT | IGS (*ycf2, trnN-GUU*) | *ycf2* |
| 43 | F | 85461; 86188 | ATTAATTGTCCGTACGAGCCGGGAGGAGCAGTAAGCGTCTGGA | IGS (*rpl20, rps12_5end*) | *rpl20* (reverse strand) |
| 43 | F | 85877; 86605 | AAATAATTTCGTTTCAATATTTCTTATTTGGAGATGTCAATTT | IGS (*rpl20, rps12_5end*) | IGS (*rpl20, rps12_5end*) |
| 43 | F | 122802; 122847 | GTGTCGACAGTGTCGATAGTGTCGATAGTGTCGATAGTGTCGA | IGS (*trnN-GUU, trnN-GUU*) | IGS (*trnN-GUU, trnN-GUU*) |
| 42 | F | 2061; 147327 | GAATCGATAAGTCATCTGGGGAGGGGACTAAATCCACAATTT | IGS (*trnN-GUU, ycf2*) | IGS (*trnN-GUU, orf42*) |
| 41 | F | 35181; 35329 | TCTCGTATTGCTATAGCATGATACCACGGAAACGGCTTTTT | IGS (*atpH, atpI*) | IGS (*atpH, atpI*) |
| 40 | F | 85419; 86146 | TAATCCGGAGAGGCTTACCGTCGCATATCAATCTCTCTCA | IGS (*rps18, rpl20*) | IGS (*rpl20, rps12_5end*) |
| 39 | F | 18501; 19678 | ATGTGGAAAAATGATACCGAAGAGTCGCGTAAATGAAGC | *ndhB* (reverse strand) | *ndhB* |
| 39 | P | 19678; 105686 | GCTTCATTTACGCGACTCTTCGGTATCATTTTTCCACAT | *ndhB* (reverse strand) | ndhB |
| 39 | F | 92998; 93616 | CAAATAAACAGTCTTTTATTTAAATAAATTTTATTATAT | IGS (psbH, petB) | IGS (*psbH, petB*) |
| 39 | F | 122810; 122855 | AGTGTCGATAGTGTCGATAGTGTCGATAGTGTCGATAGT | IGS (*trnN-GUU, trnN-GUU*) | IGS (*trnN-GUU, trnN-GUU*) |
| 38 | F | 58313; 60549 | GCAGGTACCGCCTCGGCCGGGACCATCGCAGGGAAAAC | *psaB* (reverse strand) | *psaA* (reverse strand) |
| 38 | F | 93164; 93782 | AGTTGTTATCTGATATTGCCGACTCGGCGCTGTATCAA | IGS (*psbH, petB*) | IGS (*psbH, petB*) |
| 38 | F | 151880; 151929 | TGAGAGATGAGAGATGAGAGATGAGAGATGAGAGATGA | IGS (*rrn5, trnR-ACG*) | IGS (*rrn5, trnR-ACG*) |
| 38 | F | 34391; 34862 | AAAAATTTGAGTATTTGGTAATACTAATTATTATCTAC | IGS (*atpH, atpI*) | IGS (*atpH, atpI*) |
| 38 | F | 2728; 2740 | ATTATCTTCACTATTATCTCCACTATTATCTTCACTAT | *ycf2* (reverse strand) | *ycf2* |
| 38 | P | 2740; 121460 | ATAGTGAAGATAATAGTGGAGATAATAGTGAAGATAAT | *ycf2* | *ycf2* |
| 38 | P | 2728; 121448; | ATAGTGAAGATAATAGTGGAGATAATAGTGAAGATAAT | *ycf2* (reverse strand) | *ycf2* |
| 38 | F | 94224; 94478 | AGTTTTCCGAGGCCGAGTCGCTTCTTCCCCCATTGATT | IGS (*psbH, petB*) | IGS (*psbH, petB*) |
| 38 | F | 121448; 121460 | ATAGTGAAGATAATAGTGAAGATAATAGTGGAGATAAT | *ycf2 (reverse strand)* | *ycf2* |
| 37 | P | 123798; 146607 | AAGAAGTAATATCTTTGATAAATAGAAGTAGCAAAAA | IGS (*trnN-GUU, rpl32*) | IGS (*chlL, trnN-GUU*) |
| 37 | F | 27128; 27166 | ATCCTCATCATTCGAATGCATCCTAATTATTCGAATG | IGS (*trnQ-UUG, psbI*) | IGS (*trnQ-UUG, psbI*) |
| 37 | F | 122712; 123628 | GGTACCCGAATCGTGGGAGAGGGGGGGGGGGGGGGGG | IGS (*trnN-GUU, trnN-GUU*) | IGS (*trnN-GUU, rpl32*) |
| 37 | F | 132672; 133420 | TTCTTATCAGTTGTGATTGTGACCTCGTCACGCGCTG | *ndhG* (reverse strand) | IGS (*ndhE, ndhI*) |
| 36 | F | 122691; 123607 | GCAATCCCATTTGAATGGAAGGGTACCCGAATCGTG | IGS (*trnN-GUU, trnN-GUU*) | IGS (*trnN-GUU, rpl32*) |
| 36 | F | 132518; 133262 | CAGCTAATAATCCTACCACCATATGCCACTCATTAG | *ndhB* (reverse strand) | *ndhB* |
| 36 | F | 12362; 63685 | AACCGTACGTGAGATTCCCACCTCATACGGCTCCTC | IGS (*rps12, rrn16*) | IGS (*ycf3, ycf3*) |
| 36 | F | 27391; 27407 | TCGAAGCATCCTATATTCGATGCATCCTATATTCGA | IGS (*trnQ-UUG, psbI*) | IGS (*trnQ-UUG, psbI*) |
| 36 | P | 63685; 111828 | AACCGTACGTGAGGTTCCCAACTCATACGGCTCCTC | *ycf3* (reverse strand) | IGS (*rrn16, rps12_3end*) |
| 36 | F | 59171; 61395 | AACGGCAATGGCTAAGTGGTGATGAGCAATATCAGT | *psaB* (reverse strand) | *psaA* (reverse strand) |
| 36 | F | 93174; 93792 | TGATATTGCCGACTCGGCGCTGTATCAAGAAATTAC | IGS (*psbH, petB*) | IGS (*psbH, petB*) |
| 36 | F | 35229; 35377 | CGGCGACCCAATGAATGGTTCTCAAAAAAATTATTT | IGS (atpH, atpI) | IGS (atpH, atpI) |
| 35 | F | 18462; 19639 | CTAATGAGTGGCATATCGCGGTAGGATTATTAGCT | *ndhB* (reverse strand) | *ndhB* (reverse strand) |
| 35 | P | 19639; 105729 | CTAATGAGTGGCATATCGCGGTAGGATTATTAGCT | *ndhB* (reverse strand) | *ndhB* |
| 35 | F | 34323; 34810 | TTTCCTACGTCGACCAGTGCGACTTCCTGCCTAAT | IGS (*atpH, atpI*) | IGS (*atpH, atpI*) |
| 35 | F | 34545; 34996 | TAAATGGCTTCCCAAATATTTTGTATATACTTTAT | IGS (*atpH, atpI*) | IGS (*atpH, atpI*) |
| 34 | F | 85702; 86429 | GGTTTCGGGGGGCTCATTCAAGATTATTTAGAGC | *rpl20* (reverse strand) | IGS (*rpl20, rps12_5end*) |
| 34 | P | 122712; 146774 | GGTACCCGAATCGTGGGAGAGGGGGGGGGGGGGG | IGS (*trnN-GUU, trnN-GUU*) | IGS (*chlL, trnN-GUU*) |
| 34 | F | 86709; 86895 | AGATCTCGAAGATAGATTTATATTTTAATTACAC | IGS (*rpl20, rps12_5end*) | IGS (*rpl20, rps12_5end*) |
| 34 | F | 48260; 48298 | GTAAAAGAAGAAAGACTAAGTAAAAGAAGAAAGA | IGS (*rpoB, trnD-GUC*) | IGS (*rpoB, trnD-GUC*) |
| 34 | F | 122802; 122856 | GTGTCGACAGTGTCGATAGTGTCGATAGTGTCGA | IGS (*trnN-GUU, trnN-GUU*) | IGS (*trnN-GUU, trnN-GUU*) |
| 33 | F | 18562; 19739 | ACAGAGAAAAAAGCAACGACTGGTATAGGAGAG | *ndhB* (reverse strand) | *ndhB* (reverse strand) |
| 33 | P | 19739; 105631 | CTCTCCTATACCAGTCGTTGCTTTTTTCTCTGT | *ndhB* (reverse strand) | *ndhB* |
| 33 | F | 35189; 35337 | TGCTATAGCATGATACCACGGAAACGGCTTTTT | IGS (*atpH, atpI*) | IGS (*atpH, atpI*) |
| 33 | F | 132770; 133519 | TCACCAACTCGGATATAAATTACTGAGTGAGTT | *ndhG* (reverse strand) | IGS (*ndhE, ndhI*) |
| 33 | F | 132113; 132887 | TAATATAGGGGGAGGTATCCAAACAAATTAATA | IGS (*psaC, ndhE*) | IGS (*psaC, ndhE*) |
| 32 | F | 94131; 94383 | TCGATAAGATTAAAAGATTTCCCAAGACGCTA | IGS (*psbH, petB*) | IGS (*psbH, petB*) |
| 32 | P | 2089; 123049 | TAAATCCACAATTTTAGAAAGGATCTATTCCA | IGS (*trnN-GUU, ycf2*) | IGS (*trnN-GUU, orf42*) |
| 32 | R | 131305; 131311 | CATGAATAAAAAATAATTAATAAAAAATAATT | *ndhD* | IGS (*ndhD, psaC*) |
| 32 | F | 18481; 19658 | ATATGCCACTCATTAGGGAAATGTGGAAAAAT | *ndhB* (reverse strand) | *ndhB* (reverse strand) |
| 32 | P | 19658; 105713 | ATTTTTCCACATTTCCCTAATGAGTGGCATAT | *ndhB* (reverse strand) | *ndhB* |
| 32 | F | 35276; 35424 | TAGTCTTTTGGAATGACTCATTCCAACTAAAT | IGS (*atpH, atpI*) | IGS (*atpH, atpI*) |
| 32 | F | 86814; 86999 | AAATTTATCGGCTGTGACGAATTTCTGCTTTC | IGS (*rpl20, rps12_5end*) | IGS (*rpl20, rps12_5end*) |
| 32 | F | 122266; 123182 | GCCTCGGGGGTATTCGAGGGGGGGGGGGAGGA | IGS (*ycf2, trnN-GUU*) | IGS (*trnN-GUU, trnN-GUU*) |
| 32 | P | 122266; 147222 | GCCTCGGGGGTATTCGAGGGGGGGGGGGAGGA | IGS (*ycf2, trnN-GUU*) | IGS (*ycf2, trnN-GUU*) |
| 32 | P | 122548; 146940 | AAAGAACAAAGGTCTCCGAGTGCGTATGAGAC | IGS (*trnN-GUU, trnN-GUU*) | IGS (*chlL, trnN-GUU*) |
| 31 | F | 151880; 151936 | TGAGAGATGAGAGATGAGAGATGAGAGATGA | IGS (*rrn5, trnR-ACG*) | IGS (*rrn5, trnR-ACG*) |
| 31 | F | 86778; 86964 | GAAGGTCGTTAGGTAGCTGCTTCATAATTTT | IGS (*rpl20, rps12_5end*) | IGS (*rpl20, rps12_5end*) |
| 31 | F | 131852; 132015 | AAATTTATCTGATCGGATCCCATTTGTTTCT | IGS (*psaC, ndhE*) | IGS (*psaC, ndhE*) |
| 31 | F | 131308; 131322 | ATGAATAAAAAATAATTAATAAAAAATAATT | IGS (*ndhD, psaC*) | IGS (*ndhD, psaC*) |
| 31 | F | 132616; 133364 | AAAGAAGGTTTAATCAAAAAAATGGAGAATT | *ndhG* (reverse strand) | IGS (*ndhE, ndhI*) |
| 31 | P | 28405; 64823 | AAAGGAGAGAGAGGGATTCGAACCCTCGGTA | *trnS-GCU* (reverse strand) | *trnS-GGA* |
| 31 | F | 35290; 35438 | GACTCATTCCAACTAAATTAATGGTGGCCCT | IGS (*atpH, atpI*) | IGS (*atpH, atpI*) |
| 31 | F | 85910; 86638 | ATGTCAATTTGTAGAAATAGATATGCTGTGT | IGS (*rpl20, rps12_5end*) | IGS (*rpl20, rps12_5end*) |
| 31 | F | 86746; 86932 | GTGCGGTGACATGCCGCGCTTTTCAATTTTT | IGS (*rpl20, rps12_5end*) | IGS (*rpl20, rps12_5end*) |
| 30 | F | 85323; 86049 | ATAGTCTGTCCTTCCGTTTATCTTTTCTTC | IGS (*rps18, rpl20*) | IGS (*rpl20, rps12_5end*) |
| 30 | P | 28407; 52505 | AGGAGAGAGAGGGATTCGAACCCTCGGTAC | *trnS-GCU* (reverse strand) | *trnS-UGA* |
| 30 | P | 28469; 52442 | GCCTTAAACCCCTCGGCCATCTCTCCAAGC | *trnS-GCU* (reverse strand) | *trnS-UGA* |
| 30 | F | 34471; 34924 | CAATTCATTCGATATGCTGTGACGTAGGTG | IGS (*atpH, atpI*) | IGS (*atpH, atpI*) |
| 30 | F | 85368; 86095 | GATAGATCCGCGAGTTAACTCATTCTTCAT | IGS (*rps18, rpl20*) | IGS (*rpl20, rps12_5end*) |
| 30 | F | 85377; 86104 | GCGAGTTAACTCATTCTTCATTTTATTTCT | IGS (*rps18, rpl20*) | IGS (*rpl20, rps12_5end*) |
| 30 | F | 85942; 86670 | ATACCGAAATGTACCCCCCCCCCCCCCCCG | IGS (*rpl20, rps12_5end*) | IGS (*rpl20, rps12_5end*) |
| 30 | F | 122810; 122864 | AGTGTCGATAGTGTCGATAGTGTCGATAGT | IGS (*trnN-GUU, trnN-GUU*) | IGS (*trnN-GUU, trnN-GUU*) |
| 30 | F | 2736; 2748 | CACTATTATCTCCACTATTATCTTCACTAT | *ycf2* (reverse strand) | *ycf2* (reverse strand) |
| 30 | P | 2736; 121448 | ATAGTGAAGATAATAGTGGAGATAATAGTG | *ycf2* (reverse strand) | *ycf2* |
| 30 | P | 2748; 121460 | ATAGTGAAGATAATAGTGGAGATAATAGTG | *ycf2* (reverse strand) | *ycf2* |

F, Forward (direct); R, Reverse; P, Palindromic; *, the repeat structure overlapped with the 18 bp G mononucleotide sequence.


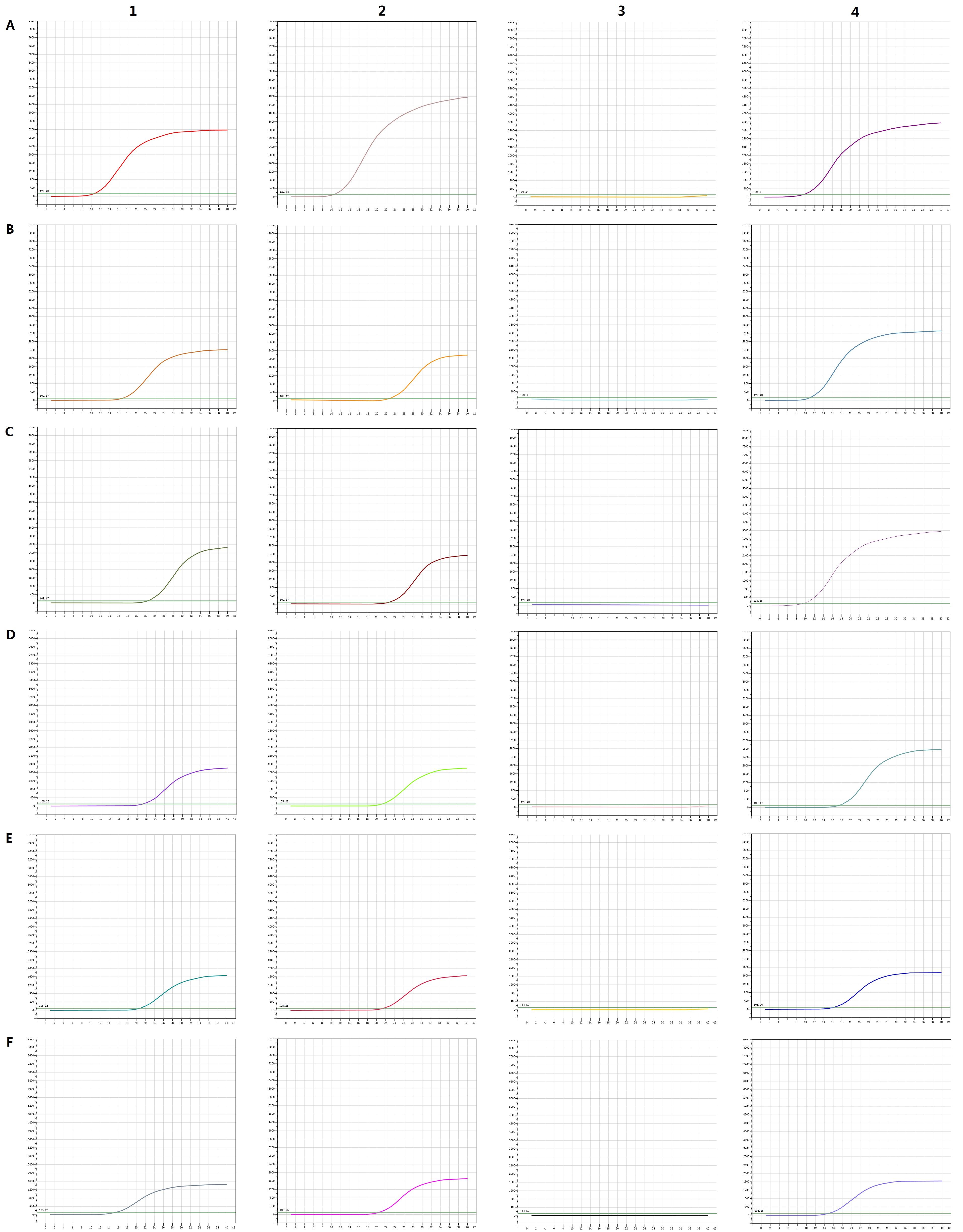


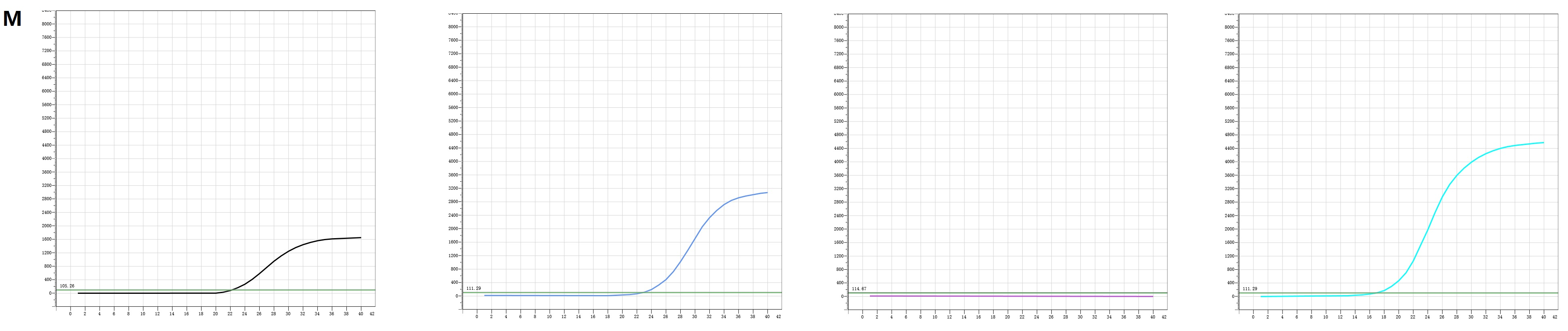

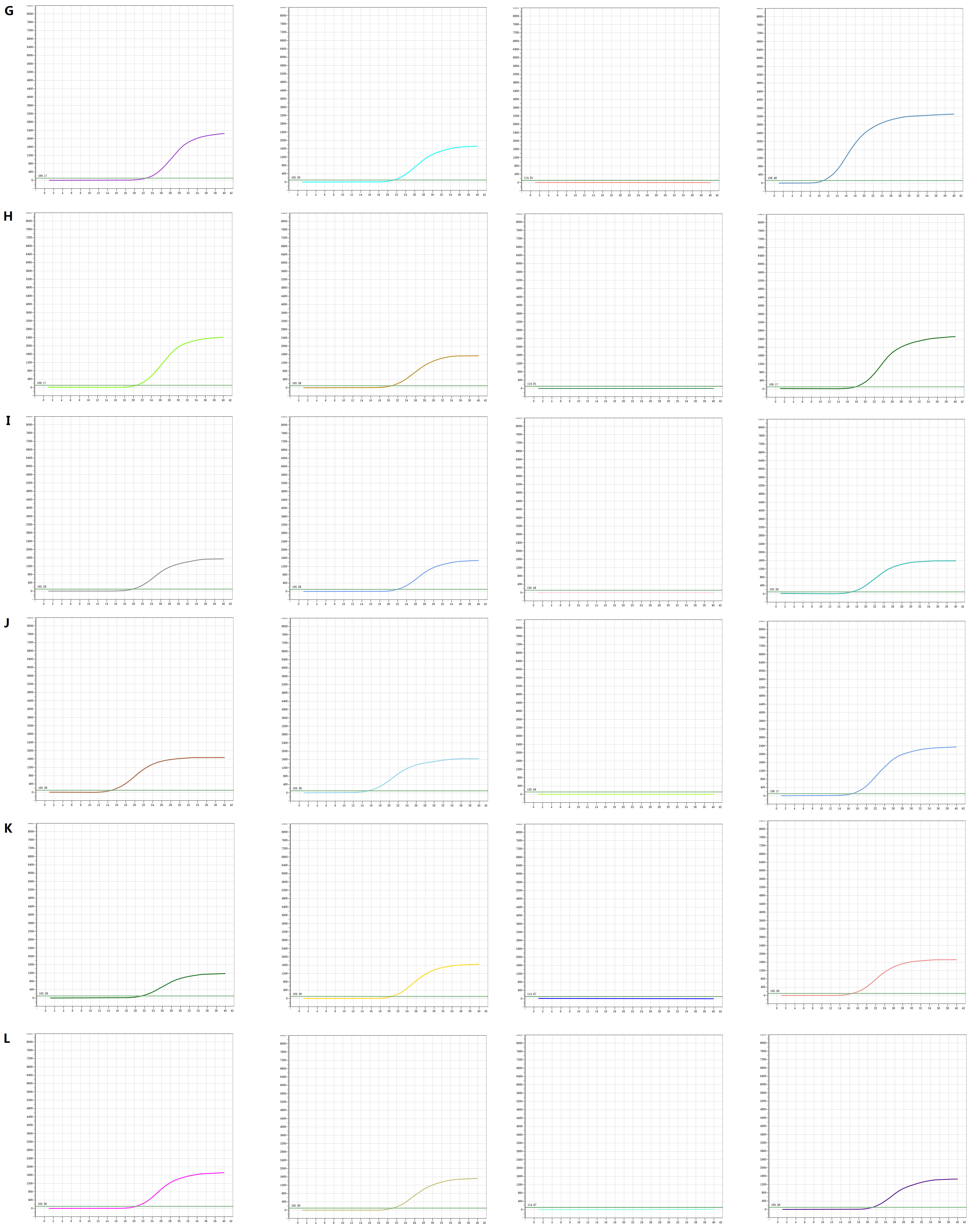


**Supplymental Fig.1 qRT-PCR tested contamination of the nuclear genome in *D. fragrans* chloroplast DNA.** 1. Nuclear specific gene *actin 6* was detected in total DNA. 2. Cp special gene *rbcL* was detected in total DNA. 3. Nuclear specific gene *actin 6* was not detected in cpDNA. 4. Cp special gene *rbcL* was detected in cpDNA. A. *D. fragrans,* B. *Triticum aestivum* (wheat)*,* C. *Arbidopsis thaliana,* D. *Microlepia strigosa,* E. *Cyrtomii fortunei*, F. *Polypodiodes amoena*, G. *Isoetes sinensis,* H. *Ceratopteris thalictroides*, I. *Phyllitis scolopendrium*, J. *Davallodes chingiae,* K. *Scutellaria sessilifolia*, L. *Thelypteris palustris* and M. *Nephrolepis biserrata*.

**Supplemental Table 4. List of RNA editing sites in protein-coding genes of *D. fragrans* cp genome.**

| **Gene** | **Strand** | **Location** | **Position** | **RNA edited site** | **Codon position transition** |
| --- | --- | --- | --- | --- | --- |
| *accD* | + | 75549..76481 | 75610 | C-U | 2 |
|  |  |  | 75611 | C-U | 3 |
|  |  |  | 75637 | C-U | 2 |
|  |  |  | 75723 | C-U | 1 |
|  |  |  | 75765 | C-U | 1 |
|  |  |  | 75868 | C-U | 2 |
|  |  |  | 75877 | A-C | 2 |
|  |  |  | 75891 | C-U | 1 |
|  |  |  | 75943 | C-U | 2 |
| *atpB* | - | 71462..72943 | 72861 | C-U | 2 |
|  |  |  | 72846 | C-U | 2 |
|  |  |  | 72618 | C-U | 2 |
|  |  |  | 72503 | C-U | 3 |
|  |  |  | 72449 | C-U | 1 |
|  |  |  | 72402 | C-U | 2 |
|  |  |  | 72396 | C-U | 2 |
|  |  |  | 72354 | C-U | 2 |
|  |  |  | 72198 | C-U | 2 |
|  |  |  | 72199 | C-U | 2 |
|  |  |  | 72144 | C-U | 2 |
|  |  |  | 72070 | C-U | 1 |
|  |  |  | 72024 | C-U | 2 |
|  |  |  | 71988 | C-U | 2 |
|  |  |  | 71922 | C-U | 2 |
|  |  |  | 71898 | C-U | 2 |
|  |  |  | 71893 | C-U | 1 |
|  |  |  | 71818 | C-U | 1 |
|  |  |  | 71766 | C-U | 2 |
|  |  |  | 71644 | C-U | 1 |
|  |  |  | 71500 | C-U | 3 |
| *atpI* | - | 35456..36202 | 36194 | C-U | 3 |
|  |  |  | 36126 | C-U | 2 |
|  |  |  | 36073 | C-U | 1 |
|  |  |  | 35920 | C-U | 1 |
|  |  |  | 35892 | C-U | 2 |
|  |  |  | 35859 | C-U | 2 |
|  |  |  | 35851 | C-U | 1 |
|  |  |  | 35758 | C-U | 1 |
|  |  |  | 35754 | C-U | 2 |
|  |  |  | 35753 | C-U | 3 |
|  |  |  | 35673 | C-U | 2 |
|  |  |  | 35670 | C-U | 2 |
|  |  |  | 35599 | C-U | 2 |
|  |  |  | 35598 | C-U | 3 |
|  |  |  | 35571 | C-U | 2 |
|  |  |  | 35540 | C-U | 2 |
|  |  |  | 35547 | C-U | 2 |
|  |  |  | 35472 | C-U | 2 |
| *ccsA* | + | 128516..129453 | 128589 | C-U | 2 |
|  |  |  | 125666 | C-U | 2 |
|  |  |  | 128763 | C-U | 2 |
|  |  |  | 128814 | C-U | 2 |
|  |  |  | 128832 | C-U | 2 |
|  |  |  | 128853 | C-U | 2 |
|  |  |  | 128901 | C-U | 2 |
|  |  |  | 128912 | C-U | 1 |
|  |  |  | 128916 | C-U | 2 |
|  |  |  | 128937 | C-U | 2 |
|  |  |  | 128973 | C-U | 2 |
|  |  |  | 128985 | C-U | 2 |
|  |  |  | 128987 | C-U | 1 |
|  |  |  | 128988 | C-U | 1 |
|  |  |  | 128990 | C-U | 2 |
|  |  |  | 129000 | C-U | 2 |
|  |  |  | 129023 | C-U | 1 |
|  |  |  | 129105 | C-U | 2 |
|  |  |  | 129114 | C-U | 2 |
|  |  |  | 129119 | C-U | 1 |
|  |  |  | 129169 | C-U | 3 |
|  |  |  | 129215 | C-U | 1 |
|  |  |  | 129227 | C-U | 1 |
|  |  |  | 129242 | C-U | 1 |
|  |  |  | 129254 | C-U | 1 |
|  |  |  | 129285 | C-U | 2 |
|  |  |  | 129293 | C-U | 1 |
|  |  |  | 129297 | C-U | 2 |
|  |  |  | 129302 | C-U | 1 |
|  |  |  | 129309 | C-U | 2 |
|  |  |  | 129260 | C-U | 2 |
|  |  |  | 129372 | C-U | 2 |
|  |  |  | 129381 | C-U | 2 |
|  |  |  | 129395 | C-U | 1 |
|  |  |  | 129398 | C-U | 1 |
|  |  |  | 129402 | C-U | 2 |
| *clpP* | - | 88065..89930 | 89130 | C-U | 2 |
|  |  |  | 89034 | C-U | 2 |
|  |  |  | 88923 | C-U | 2 |
|  |  |  | 88915 | C-U | 1 |
|  |  |  | 88914 | C-U | 2 |
|  |  |  | 88913 | C-U | 3 |
|  |  |  | 88228 | C-U | 2 |
|  |  |  | 88225 | C-U | 2 |
|  |  |  | 88133 | C-U | 1 |
|  |  |  | 88132 | C-U | 2 |
|  |  |  | 88099 | C-U | 2 |
|  |  |  | 88093 | C-U | 2 |
| *matK* | - | 21727..23232 | 22982 | G-A | 2 |
|  |  |  | 22970 | G-A | 2 |
|  |  |  | 22926 | C-U | 1 |
|  |  |  | 22823 | C-U | 2 |
|  |  |  | 22822 | A-G | 3 |
|  |  |  | 22818 | C-U | 1 |
|  |  |  | 22777 | C-U | 3 |
|  |  |  | 22982 | G-A | 2 |
|  |  |  | 22970 | G-A | 2 |
| *ndhB* | - | 19009..21514 | 18211 | A-G | 3 |
|  |  |  | 18197 | C-U | 2 |
|  |  |  | 18190 | A-C | 3 |
|  |  |  | 18184 | C-U | 3 |
|  |  |  | 18183 | U-C | 1 |
|  |  |  | 18178 | G-A | 3 |
|  |  |  | 18171 | C-U | 1 |
|  |  |  | 18164 | C-U | 2 |
|  |  |  | 18154 | G-U | 3 |
|  |  |  | 18092 | C-U | 2 |
|  |  |  | 18088 | U-A | 3 |
|  |  |  | 18084 | U-C | 1 |
|  |  |  | 18077 | C-U | 2 |
|  |  |  | 18073 | U-C | 3 |
|  |  |  | 18072 | C-U | 1 |
|  |  |  | 18070 | G-U | 3 |
|  |  |  | 18066 | G-C | 1 |
|  |  |  | 18061 | C-U | 3 |
|  |  |  | 18027 | C-U | 3 |
| *ndhD* | - | 129802..131310 | 131292 | C-U | 1 |
|  |  |  | 131271 | C-U | 1 |
|  |  |  | 131249 | C-U | 2 |
|  |  |  | 131198 | C-U | 2 |
|  |  |  | 131154 | C-U | 1 |
|  |  |  | 131146 | C-U | 3 |
|  |  |  | 131089 | C-U | 3 |
|  |  |  | 131036 | C-U | 2 |
|  |  |  | 131003 | C-U | 2 |
|  |  |  | 130965 | C-U | 1 |
|  |  |  | 130958 | C-U | 2 |
|  |  |  | 130871 | C-U | 2 |
|  |  |  | 130845 | C-U | 1 |
|  |  |  | 130826 | C-U | 2 |
|  |  |  | 130802 | C-U | 2 |
|  |  |  | 130754 | C-U | 2 |
|  |  |  | 130746 | C-U | 1 |
|  |  |  | 130655 | C-U | 2 |
|  |  |  | 130627 | G-A | 3 |
|  |  |  | 130581 | C-U | 1 |
|  |  |  | 130547 | C-U | 2 |
|  |  |  | 130103 | C-U | 2 |
|  |  |  | 129878 | C-U | 1 |
|  |  |  | 129852 | C-U | 1 |
| *ndhF* | - | 123919..126174 | 130581 | C-U | 1 |
|  |  |  | 130547 | C-U | 2 |
|  |  |  | 130103 | C-U | 2 |
|  |  |  | 126111 | C-U | 1 |
|  |  |  | 125946 | C-U | 1 |
|  |  |  | 125861 | C-U | 2 |
|  |  |  | 125841 | C-U | 1 |
|  |  |  | 125827 | C-U | 3 |
|  |  |  | 125818 | C-U | 3 |
|  |  |  | 125804 | C-U | 2 |
|  |  |  | 125762 | C-U | 2 |
|  |  |  | 125750 | C-U | 2 |
|  |  |  | 125718 | C-U | 1 |
|  |  |  | 125700 | C-U | 1 |
|  |  |  | 125676 | C-U | 1 |
|  |  |  | 125577 | C-U | 1 |
|  |  |  | 125477 | C-U | 2 |
|  |  |  | 125471 | C-U | 2 |
|  |  |  | 125363 | C-U | 2 |
|  |  |  | 125345 | C-U | 2 |
|  |  |  | 125344 | C-U | 3 |
|  |  |  | 125261 | C-U | 2 |
|  |  |  | 125258 | C-U | 2 |
|  |  |  | 125174 | C-U | 2 |
|  |  |  | 125168 | C-U | 2 |
|  |  |  | 125105 | C-U | 2 |
|  |  |  | 125102 | C-U | 2 |
|  |  |  | 125096 | C-U | 2 |
|  |  |  | 124976 | C-U | 2 |
|  |  |  | 124959 | C-U | 1 |
|  |  |  | 124910 | C-U | 2 |
|  |  |  | 124897 | C-U | 2 |
|  |  |  | 124832 | C-U | 2 |
|  |  |  | 124626 | C-U | 1 |
|  |  |  | 124595 | C-U | 2 |
|  |  |  | 124433 | C-U | 2 |
|  |  |  | 124141 | C-U | 3 |
|  |  |  | 124138 | C-U | 3 |
|  |  |  | 124055 | C-U | 2 |
|  |  |  | 124052 | C-U | 2 |
|  |  |  | 123962 | C-U | 2 |
|  |  |  | 130581 | C-U | 1 |
| *ndhG* | - | 133378..133971 | 133924 | G-A | 3 |
|  |  |  | 133882 | C-U | 3 |
|  |  |  | 133745 | C-U | 2 |
|  |  |  | 133457 | C-U | 2 |
|  |  |  | 132813 | C-U | 3 |
|  |  |  | 132812 | A-G | 1 |
|  |  |  | 132811 | G-A | 2 |
|  |  |  | 132806 | C-U | 1 |
|  |  |  | 132805 | G-A | 2 |
|  |  |  | 132804 | U-C | 3 |
|  |  |  | 132791 | U-G | 1 |
|  |  |  | 132767 | U-G | 1 |
|  |  |  | 132756 | A-G | 3 |
|  |  |  | 132709 | C-U | 1 |
|  |  |  | 132710 | U-C | 2 |
|  |  |  | 132697 | A-G | 2 |
|  |  |  | 132694 | U-A | 2 |
|  |  |  | 132687 | C-U | 3 |
|  |  |  | 132672 | G-A | 3 |
|  |  |  | 132665 | C-U | 1 |
|  |  |  | 132664 | C-U | 2 |
|  |  |  | 132663 | U-C | 3 |
|  |  |  | 132659 | U-C | 1 |
|  |  |  | 132656 | U-C | 1 |
|  |  |  | 132652 | A-G | 2 |
|  |  |  | 132651 | A-C | 3 |
|  |  |  | 132648 | U-C | 3 |
| *petB* | + | 92963..95334 | 94771 | C-U | 1 |
|  |  |  | 94798 | C-U | 1 |
|  |  |  | 94805 | C-U | 2 |
|  |  |  | 94826 | C-U | 2 |
|  |  |  | 94837 | C-U | 1 |
|  |  |  | 94838 | C-U | 2 |
|  |  |  | 94840 | C-U | 1 |
|  |  |  | 94843 | C-U | 1 |
|  |  |  | 94868 | C-U | 2 |
|  |  |  | 94886 | C-U | 2 |
|  |  |  | 94967 | C-U | 2 |
|  |  |  | 95042 | C-U | 2 |
|  |  |  | 95051 | C-U | 2 |
|  |  |  | 95174 | C-U | 2 |
|  |  |  | 95261 | C-U | 2 |
|  |  |  | 95264 | C-U | 2 |
|  |  |  | 95267 | C-U | 2 |
|  |  |  | 95282 | C-U | 2 |
|  |  |  | 95305 | C-U | 1 |
| *petD* | + | 95508..96629 | 96445 | C-U | 2 |
|  |  |  | 96487 | C-U | 2 |
|  |  |  | 96488 | C-U | 3 |
|  |  |  | 96542 | C-U | 3 |
|  |  |  | 96543 | C-U | 1 |
|  |  |  | 96559 | C-U | 2 |
|  |  |  | 96574 | C-U | 2 |
|  |  |  | 96610 | C-U | 2 |
|  |  |  | 96616 | C-U | 2 |
| *petG* | + | 83252..83368 | 83260 | C-U | 2 |
|  |  |  | 83283 | C-U | 2 |
|  |  |  | 83289 | C-U | 2 |
|  |  |  | 83331 | C-U | 2 |
| *petL* | + | 83002..83097 | 83025 | C-U | 3 |
|  |  |  | 83048 | C-U | 2 |
|  |  |  | 83051 | C-U | 2 |
| *psaI* | + | 76822..76932 | 76834 | C-U | 1 |
|  |  |  | 76898 | C-U | 2 |
| *psbB* | + | 90390..91916 | 90402 | C-U | 1 |
|  |  |  | 90505 | C-U | 2 |
|  |  |  | 90582 | C-U | 1 |
|  |  |  | 90691 | C-U | 2 |
|  |  |  | 90692 | C-U | 3 |
| *psbE* | - | 82005..82256 | 82227 | C-U | 3 |
|  |  |  | 82177 | C-U | 2 |
|  |  |  | 82168 | C-U | 2 |
|  |  |  | 82146 | C-U | 2 |
|  |  |  | 82127 | C-U | 2 |
| *psbF* | - | 81870..81989 | 81910 | C-U | 2 |
|  |  |  | 81909 | C-U | 3 |
| *psbL* | - | 81732..81848 | 81811 | C-U | 2 |
|  |  |  | 81791 | C-U | 1 |
|  |  |  | 81760 | C-U | 2 |
|  |  |  | 81757 | C-U | 2 |
| *rpoA* | - | 96967..97968 | 97904 | C-U | 2 |
|  |  |  | 97844 | C-U | 2 |
|  |  |  | 97811 | C-U | 2 |
|  |  |  | 97706 | C-U | 2 |
|  |  |  | 97685 | C-U | 2 |
|  |  |  | 97646 | C-U | 2 |
|  |  |  | 97607 | C-U | 2 |
|  |  |  | 97523 | C-U | 2 |
|  |  |  | 97484 | C-U | 2 |
|  |  |  | 97442 | C-U | 2 |
|  |  |  | 97376 | C-U | 2 |
|  |  |  | 97373 | C-U | 2 |
|  |  |  | 97367 | C-U | 2 |
|  |  |  | 97343 | C-U | 2 |
|  |  |  | 97292 | C-U | 2 |
|  |  |  | 97124 | C-U | 2 |
|  |  |  | 97087 | C-U | 3 |
|  |  |  | 97058 | C-U | 2 |
| *rpoB* | - | 44463..47678 | 47450 | C-U | 1 |
|  |  |  | 47401 | C-U | 2 |
|  |  |  | 47387 | C-U | 1 |
|  |  |  | 47365 | C-U | 2 |
|  |  |  | 47254 | C-U | 2 |
|  |  |  | 47243 | C-U | 1 |
|  |  |  | 47224 | C-U | 2 |
|  |  |  | 46825 | C-U | 2 |
|  |  |  | 45098 | U-C | 1 |
|  |  |  | 45097 | C-G | 2 |
|  |  |  | 45043 | C-U | 2 |
|  |  |  | 44965 | C-U | 2 |
|  |  |  | 47450 | C-U | 1 |
|  |  |  | 47401 | C-U | 2 |
|  |  |  | 47387 | C-U | 1 |
|  |  |  | 47365 | C-U | 2 |
| *rpoC1* | - | 41667..44412 | 42286 | C-U | 2 |
|  |  |  | 42254 | U-C | 1 |
| *rps2* | - | 36369..37085 | 36925 | C-U | 2 |
|  |  |  | 36873 | C-U | 3 |
|  |  |  | 36872 | C-U | 1 |
|  |  |  | 36871 | C-U | 2 |
|  |  |  | 36867 | C-U | 2 |
|  |  |  | 36692 | U-C | 1 |
|  |  |  | 36691 | C-G | 2 |
|  |  |  | 36449 | C-U | 1 |
|  |  |  | 36424 | C-U | 2 |
|  |  |  | 36925 | C-U | 2 |
| *rps8* | - | 99097..99495 | 99428 | C-U | 2 |
|  |  |  | 99320 | C-U | 2 |
|  |  |  | 99179 | C-U | 2 |
|  |  |  | 99113 | C-U | 2 |
|  |  |  | 99111 | C-U | 1 |
| *rps14* | - | 138145..138414 | 57432 | C-U | 2 |
| *ycf3* | - | 62634..64505 | 63533 | C-U | 2 |
|  |  |  | 63479 | C-U | 2 |
|  |  |  | 63465 | C-U | 1 |
|  |  |  | 63464 | C-U | 2 |
|  |  |  | 63461 | C-U | 2 |
|  |  |  | 63423 | C-U | 1 |
|  |  |  | 62704 | C-U | 2 |
|  |  |  | 62686 | C-U | 2 |
|  |  |  | 62672 | C-U | 1 |
|  |  |  | 62644 | C-U | 2 |
|  |  |  | 63533 | C-U | 2 |
| *atpA* | - | 30618..32141 | 32004 | C-U | 3 |
|  |  |  | 31981 | C-U | 2 |
|  |  |  | 31945 | C-U | 2 |
|  |  |  | 31912 | C-U | 2 |
|  |  |  | 31860 | A-G | 3 |
|  |  |  | 31753 | C-U | 2 |
|  |  |  | 31585 | C-U | 2 |
|  |  |  | 31501 | C-U | 2 |
|  |  |  | 31366 | C-U | 2 |
|  |  |  | 31358 | C-U | 1 |
|  |  |  | 31309 | C-U | 2 |
|  |  |  | 31262 | C-U | 1 |
|  |  |  | 31105 | C-U | 2 |
|  |  |  | 30793 | C-U | 2 |
|  |  |  | 30712 | C-U | 2 |
|  |  |  | 30658 | C-U | 2 |
|  |  |  | 30637 | C-U | 2 |
|  |  |  | 30636 | C-U | 3 |
| *rps16* | - | 23884..24928 | 23904 | U-C | 1 |


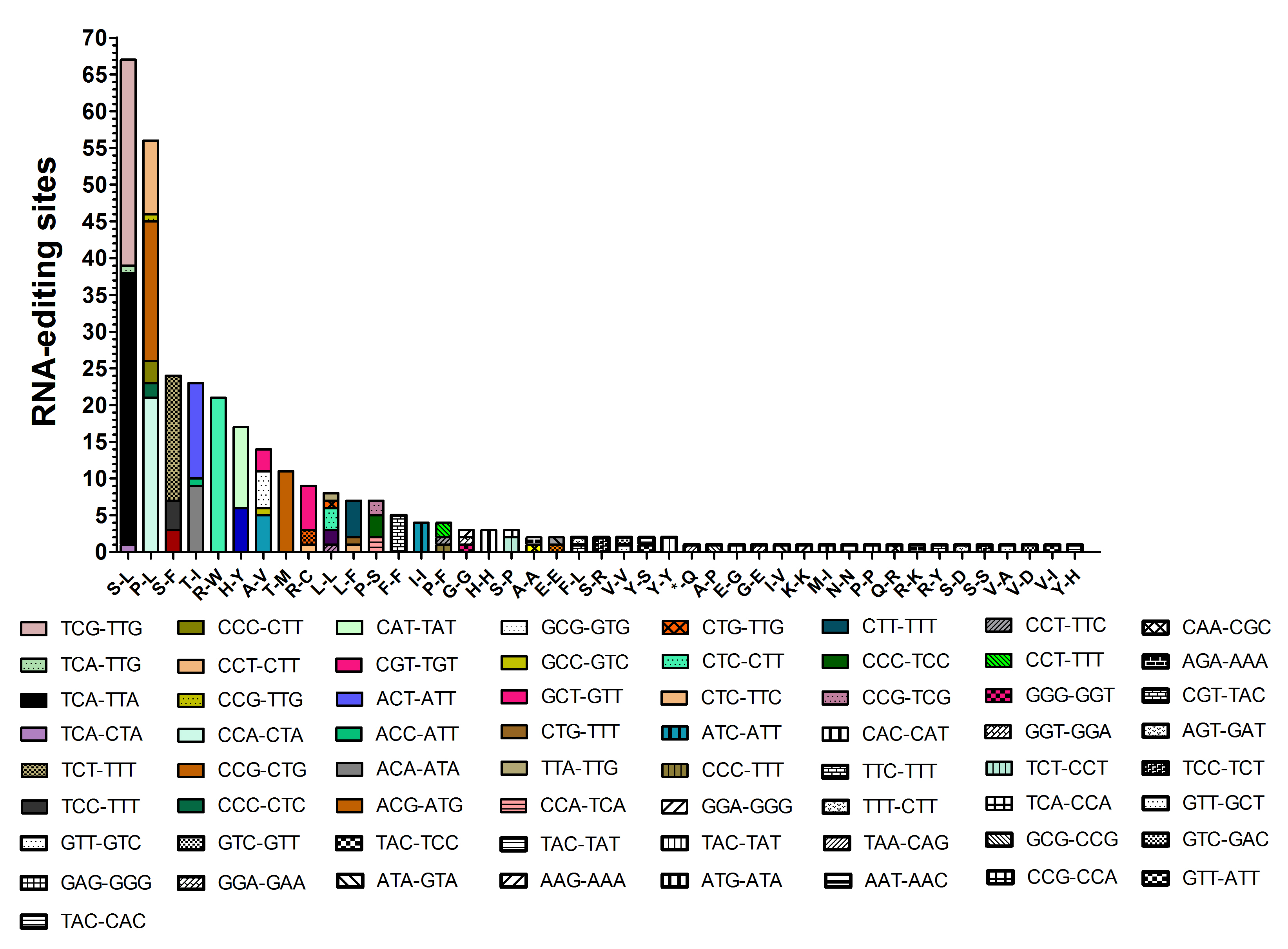


**Supplymental Figure 2. Statistics of RNA editing sites and codon transformation in *D. fragrans* cp genome.**

**Supplemental Table 5. Primer pairs for transcript validation of RNA editing sites in protein-coding genes.**

| **Gene** | **Forward primer** | **Reverse primer** |
| --- | --- | --- |
| *accD* | AGGAAACAACAGATGAGGAGATTCCT | CCGAGCAAATTCAATTTTTTTTATAAGGAGCTGA |
| *atpA* | GAATCTGCAACCGTATTACTTACTTCTCTT | TCTGACAGAAATGGTAATAAACATTCGACCTG |
| *atpB* | AGCCATCTCTTTTACTGACCCTCCAC | CCGAAATAGTTATGAAAACCAGTTCTCTCTC |
| *atpF* | AATCTTGCAAAATCTTCCAGGAGGGAAAGGA | GAGATGATAAGGTGGGAACTAGTAGTGAAAT |
| *ahpH* | AACGTAAATAAATTAGGAGGAGACT | ACAAGTTAAACAAATGGATTCGCAA |
| *atpI* | AGATAAATTTCCTCATTGATAGGGGGGTATT | CGTAGTCTTTTGCAACGACTCATTCCAACT |
| *ccsA* | CCTTCATTACGATGTATACCTATATGGAACG | GTTCCTATATTAGCCATCCGTAGTTGTG |
| *chlL* | CGGGAGAAATGAACACGAATTGGAGCTTCGCGA | TATACGCAAAGAAGCAGCCTCTCTAGTATCAAA |
| *clpP* | CTCTTCTAGTAGTTTTTTCATGCCAATTGGTG | CTTATCGAATCTTCAAGCTGACGCG |
| *matK* | GTGATGAAAATAACTTCTGGATCCCCTC | TTTATTGGACTTGTATCTCCAATGAAACAAATG |
| *ndhA* | GAGGTTGATCGTTGAAATGGCAACTG | GGGAGGCTAGCTTATTAGCAAAAGTTG |
| *ndhB* | CACTACGAATTACACTAATTTTACGTTCCC | TTCCCCCGTGAAATTGATGGAGAA |
| *ndhD* | GATTTTTCCTACACCTTCACCTCATAGC | TTTATTCATGATGAGTAATGTACCTCGGCT |
| *ndhF* | GCGGTGAAATTGCCGATATTCCC | GGAAAATCAACAACTAGCTGCATCGGG |
| *ndhG* | TAAGTACTTGTGTCCAACGAATCTATCTAAA | AGGAAAGAAGGTTTAATCAAAAAAATGGAGAA |
| *petA* | AACCCGTTTGTATAAAGACTTGAG | GCCACGGAAGAGATAACCAGAAAAAGGG |
| *petB* | CCTATCTCACTAAGGTATATGATTGGTTCGA | AATCGTGAAGGACCCGGAATGC |
| *petD* | GTTTTGAGAGCAAAATTAGCTAAAGG | GGAGGTAGAAAACTACTAGAAAAGCCCC |
| *petG* | CTAGAATGGTTGAAGCATCACTATCGG | CTATCGAATATCTAATTGATCGCCGCG |
| *petL* | CGGTATCCAAATTTATGCTCACTCTATTAA | CCAAATCACAGAATTTGAATCTTGTTCAATCC |
| *psaI* | GTAGTTGAGGTAATTTCATCATGGCAGC | GATTTCATCCCGTTCGATATATAGAAATGGG |
| *psbB* | CCAAGAAAGGGGTTTCTCACGGG | CCTGATAAGGAGAGGAGTTCGATAGGG |
| *psbC* | CTGGATGGCAGCTCAGGATCAGCCT | TTTTAATTAAGGGGTGTCATGGAAAGAACGGG |
| *psbE* | CATTGGTATTGGTTTCTCCTAAAAGAATTTGG | CGGAGCTGAACATGTCTGGGA |
| *psbL* | TAATTGAAGAAGTAATTAGAAAAAGGAGCGGC | GCTCCGAATTTACGACACAACCG |
| *psbM* | CCAATTGATGATTTGAACTACCAA | TAGTTATCGATTACTAGTTATTCTGCG |
| *petN* | GAGAGAGTAAATAGATAGGAATTACAAC | CGGCTAGCCGTTTGTTACAACCC |
| *rpl14* | ACCTACTATTTTTCCCGATTACCA | CTGATTATGCTAAATTATTTTAATCACTTATTTCT |
| *rpl23* | GAATTGAAGTAAAATGGACGAAGCGAGAAGGATA | GATGGTAAAACAAACTTCCCTTAAGTATTAG |
| *rpoA* | CCTACTGATTGACATGCAACTGATTTTTG | GGGGATTCCTTTATGCTCACGTG |
| *rpoB* | CCTCGAGTGTAGGCTTCGACAGC | CGGATACCCTCAAAATCATCGTAGATTGC |
| *rpoC1* | TTCATCGAACTAATTATCAACAGCTTCGG | TGTTGTCATATCCGATGCGGACG |
| *rps3* | ATAATTTAATATTGAGTTGAGGAGAAATAGA | TCAGGTAGTTTTTATTTCATTGGGGATCACC |
| *rps4* | TCGCTACCGAGGACCTCGCCTGAG | ATGATCACTAAGCTTTGCGGGAGTAATACT |
| *rps7* | ATATTCGTAAACTCGTGTTACATCGGAGCAGT | GTGGATTGGAACGAATCCGAAACTAACGGAAAT |
| *rps8* | GATAACTACAAAAACTACTGATTGATATTTC | CAGTTGAAAAGAAGTTTCCGAATTACCAGA |
| *rps15* | CGATTTGAGATGGAGCAAGCAATCGTAATTATTATCA | CGATATGTCGAAATATAAAACCTCTGTTCACCCT |
| *rps16* | TGTATCAGAGATTGATAAATCTTATCGA | CTTCTTCGTCTTCAACAAGTATCACGAT |
| *rps18* | GTATATTGGAATGTTTATTGGTAGTCACAAA | GTCAAATTGAGAAATCCCCCAGAATTCA |
| *rps19* | ATATTAAGCAGGGGGTTAATCGGC | CGTTTTTCATGAAGTGTAATTTCCTAATCATCG |
| *ycf1* | GCTTCATACCCCCTTGCTCCTGCTAATTTC | GTGGAATGTCAATTCCGTACATCAAAAAGACT |
| *ycf3* | GCGGTGCGGAAAAAAACAAGGAGTCGAA | CCTTTACTAATTCACTAATTAACTAAGCG |
